# Reward and punishment in a team contest

**DOI:** 10.1371/journal.pone.0236544

**Published:** 2020-09-17

**Authors:** Florian Heine, Martin Strobel

**Affiliations:** 1 Tilburg University, Tilburg School of Economics and Management, Tilburg, The Netherlands; 2 Maastricht University, Department of Economics, Maastricht, The Netherlands; Heidelberg University, GERMANY

## Abstract

A team contest entails both public good characteristics within the teams as well as a contest across teams. In an experimental study, we analyse behaviour in such a team contest when allowing to punish or to reward other team members. Moreover, we compare two types of contest environment: One in which two teams compete for a prize and another one in which we switch off the between-group element of the contest. We find that reward giving, as opposed to punishing, induces higher contributions to the team contest. Furthermore, expenditures on rewarding other co-players are significantly higher than those for punishing.

## Introduction

Social interactions are usually more complex than pictured by our simple models of cooperation or competition. One class of examples are situations in which individuals compete in groups. They bundle their resources or complement their competences to be more powerful than individual competitors or other groups. Exerting effort in these competitions comes at an irreversible cost to the individual, while the gains reaped from the group’s success benefit every group member alike, irrespective of their individual efforts. An example are architectural offices competing to win the public tender for a new opera house. Each office member’s effort is costly but contributes to the office’s chances to win the tender. The efforts of the losing teams are wasted. Other examples are sport teams competing for a trophy; or workforces of companies who team up to achieve better outcomes in collective wage negotiations. The same is done by companies. The burden of strikes (as one of the main means) is carried by a fraction of employees or employers only, but the results can be enjoyed by all members of the corresponding group. Strategically, all these situations entail aspects of dilemmas within groups as well as of contests across groups. Eventually they can be modeled as complex coordination games.

Most studies so far have investigated isolated groups, abstracting from the element of between-group competition. For those dilemma situations there exists a vast literature in philosophy, economics, and psychology discussing the role of institutions to overcome dilemma problems within a group (e.g. [1–4]). A simple form of institutionalising would be social ostracism or mobbing on the one hand, or promotion or appraisal on the other—mechanisms we frequently use in the absence of complete contracts. Earlier studies suggest that the installation of these systems can significantly reduce free-rider problems. In these studies, mobbing or appraisal are represented by the opportunity to punish or reward co-players, respectively (e.g. [5–10]). The results are not yet fully conclusive, but it seems that punishment works better than rewarding and therefore they give a first indication for policy advice: Use the stick rather than the carrot [11, 12].

Embedding a group dilemma situation into a contest with another group changes the nature of the game, and brings about interesting features. Firstly, contributions to the group still have positive external effects onto other group members, but from the point of view of a social planner, high effort levels are not desirable in these kind of contests, as exerting effort is costly. A recurring issue in experimental studies on contest games is players’ persistent tendency to over-contribute and the ensuing effects on efficiency (cf. [13, 14]). In an experimental study, Abbink et al. [15] find that allowing for the opportunity to punish other group members in a contest game renders expenditure levels that are as far as 60% above those in the control treatment.

Secondly, a conflict or a competition with another team may increase in-group favouritism and out-group spite, a desire to benefit players from the own team and harm those from the competing team [16, 17]. This is often referred to as parochial altruism [18–20]. There are various psychological motivations for this phenomenon. One considers cognitive factors: Doise [21] argues that being categorised in competitive teams, an anticipatory-justification process is active, devaluing one’s antagonists. Another one considers motivational factors: According to Tajfel [22], people desire to compare the in-group in a favourable way towards the out-group. The differentiation between an own group and an out group creates a favouring atmosphere towards the own group from the very beginning [23]. This can lead to the “ultimate attribution error” [24], where positive actions of out-group members are explained away as exceptions to the rule, for example. Pettigrew [24] mentions that this pattern of attributions is stronger if groups are in conflict with each other.

Attitudes towards others can be interpreted as weights in an other-regarding preference model (for details see next section). A shift in these weights might also change the players’ preference for either rewarding or punishing other team mates’ actions. A more positive in-group attitude makes punishment for own group members more costly while rewarding becomes cheaper. These effects seem to make the carrot a more appropriate tool for fostering contributions than the stick.

Our project brings together the two interaction paradigms of dilemmas and contests on the one hand, with reward and sanctioning systems on the other. Experimental research in this field has mainly focused on subsets of these concepts. In related experiments by Sefton et al. [9], agents are not in a competitive situation and in Abbink et al. [15], participants have no opportunity to give rewards.

Our purpose is to investigate whether we observe recognisable patterns as in the behaviour of dilemmas or contests. More specifically, we consider the effect that the presence of another competing group has with respect to contribution, punishment or rewarding. To this end, we make the presence of a competing group a systematic treatment variable. We match the experimental treatments of our group contest game with an equivalent set of treatments without a contest against another group. In the former set of treatments there exists an explicit distinction between an in-group and an out-group, while in the latter treatments, this is not the case. Without this distinction, “people are unlikely to attend to the fact that they are interacting with in-group members” [17].

In line with the vast majority of research on rent-seeking or contest games, we find an over-contribution across all our treatments, as compared to the Nash-equilibrium (cf. [13, 14, 25]). This leads to an inefficient outcome and rent over-dissipation. Furthermore, this over-contribution is more pronounced in the treatments with opponent group. Next to that, participants distribute more reward than punishment (comparing between and within treatments). This gives a new perspective on group dynamics in interactive economic games. We argue that the environment a group faces, has significant influence on its social dynamics and preferences within the group.

The remainder of this article is structured as follows: First, we describe the setup of the experiment and derive some hypotheses from a very simple other-regarding utility model; the Results Section contains our results and interpretation; followed by concluding comments and some suggestions for further research.

## Experimental design

The central issue we investigate in this experimental study is the comparison of reward and punishment in team contests and the consequences for contribution levels. On that account we employ two different environments:

1. A team contest game in which two teams compete for a prize. Players’ costly efforts have positive externalities, i.e. they increase the own team’s winning chance.
2. A game with the same local positive externalities, but without the dynamic contest against human opponents.

Switching off the contest in the second environment affects not just one but multiple aspects. *First of all*, we remove the antagonist team. *Secondly*, and as a consequence, we also remove the strategic uncertainty that the antagonist team exerts on players. And *lastly* we remove the more subtle cues in the instructions that point towards in-group behaviour (e.g. we entirely avoid the term “team” in the environment without contest). An alternative setup could have incorporated some form of randomisation on the side of the opponent party in the latter environment as for example in Cox [26]. We decided against this—and in favour of a static opponent contribution level—to create a setup that excludes all elements emanating from competing with another real group. No opponent group also means that there is no scope for strategic interaction with the other group. In the dynamic environment, two teams may collude, reciprocate, imitate or threaten each other; all of which are dynamics we would like to switch off for the latter environment.

All other aspects were identical between the environments. Rather than in the individual aspects we were interested in the overall effect that contests have on the institutions that group members use to encourage / discipline their peers. We execute this study as controlled computerised laboratory experiment, as this allows us to neatly design the environment and administer the appropriate institutional system.

All experiments were conducted with the informed consent of healthy adult subjects who were free to withdraw from participation at any time. Only individuals who voluntarily entered the experiment recruiting database were invited, and informed consent was indicated by electronic acceptance of an invitation to attend an experimental session. The experiments were conducted following the procedures established by Maastricht University’s Behavioral and Experimental Economics Laboratory (BEElab). Our study was approved in an open peer review meeting that is mandatory for all scholars wishing to use the BEElab facilities.

Players are randomly sorted in groups of four. In the field, agents repeatedly interact with the same set of others; hence also throughout the experiment participants play with the same players both in the own and in the potential opponent group. Each player also keeps the same label throughout the experiment. The experiment is conducted over 15 rounds. This and all other features of the experiment are disclosed and are commonly known to the participants.

The experiment is set up in a 2 × 4 design. The first dimension varies the presence of an opponent group (contest versus non-contest), the second dimension varies the possibility for group members to react on each others contribution (none, punishment, reward, reward & punishment). In the treatments with opponent group, every group *K* of four players competes against another group *M* of four players. They compete by buying lottery tickets for their own group. Then one ticket will be drawn by the computer and this group wins a fixed prize. For the treatments without opponent group, the other group’s tickets are replaced by a fixed number of 25 blank tickets which do not result in rent payments if drawn. Every round is partitioned into three distinct stages:

1. Each player receives an endowment (budget) of *B* = 100 tokens and decides how much of it to invest in order to buy lottery tickets named to her team. The price for a ticket is one token. Investment of player *k* ∈ *K* of group *K* is labeled *v*_*k*_ and *m* ∈ *M* of group *M* is *v*_*m*_. All tokens that a player does not invest will be added to her private account.
2. After the investment phase the tickets of both groups are pooled and one ticket is drawn randomly. For the treatments without opponent, group *M*’s tickets are replaced by a total of 25 blank tickets, which is known to all players. As will be discussed below this represents the Nash-equilibrium group contribution. Members of the winning group each receive a prize of *z* = 100 tokens, the other group gets nothing. In the non-contest treatments, if a blank ticket is drawn, the prize is forfeited. The design resembles a Tullock contest as in [27, 28]. The winning probability for the contest environment is:
  $$\begin{array}{c} p_{K}({\left(v_{k}\right)}_{k\in K},{\left(v_{m}\right)}_{m\in M})=\{\begin{array}{cc} \frac{\sum_{k\in K}v_{k}}{\sum_{k\in K}v_{k}+\sum_{m\in M}v_{m}} & \;\text{if}\underset{i\in K\cup M}{\text{max}}\{v_{i}\}>0\\ \frac{1}{2} & \;\text{otherwise} \end{array} \end{array}$$ (1)
  The winning probability for the non-contest environment is:
  $$\begin{array}{c} p_{K}({\left(v_{k}\right)}_{k\in K})=\frac{\sum_{k\in K}v_{k}}{\sum_{k\in K}v_{k}+25} \end{array}$$ (2)
  where *p*_*K*_ is the probability that group *K* wins (over group *M*).
  This design with probabilistic contest success function captures the stochastic elements inherent to applications we intend to model here (i.e. R&D races, sports competition, and other types of rent-seeking competition). In these kinds of applications, the winner will not necessarily be the party that puts in marginally more effort; there is frequently some element of luck involved.
3. The players get to know whether their group has won or not and how much each of the others in their group contributed. They also get to know the number of lottery tickets bought by the opponent team, but not the opponent players’ individual contributions (if applicable). As well, participants get to know, what their probability of winning was, which takes into account the sum of contributions of the opponent group / blank tickets. They now receive another *F* = 50 tokens (response/Feedback tokens), which they can either keep in their own account or spend to give response to their own group members. Throughout the article we regularly use the expression response when referring to the reward and punishment mechanism in order to ease the reading flow. As well, we make use of the term sanctioning as synonymous term for punishment. We employ four treatments, each played with and without opponent group.
  *[Baseline:]* Players receive the aforementioned 50 response tokens at the end of each round to be added to their account directly. As in this treatment there is no further interaction between the players, these tokens are presented as extra tokens.
  *[Reward:]* Players can reward co-players in their own group. For this, they assign response tokens to one or more players of their own group. Each response token player *l* assigns to another player *k* is added to *k*’s account. However, *l* can also keep response tokens for herself and let them be added to her own account. Participants cannot save tokens to be spent in upcoming rounds.
  *[Punish:]* This is similar to the reward treatment, but with punishment instead. Consider again two players, *l* and *k*. *l* can assign deduction points to any other player of her own group, say *k*. For this, the same amount of response tokens spent by *l*, is deducted from *k*’s account. In this treatment, it is possible that a player gets punished such that her round payoff would theoretically turn negative. However, we make participants aware that in such a case the targeted player’s outcome would be set to zero for that round. In the experiment such a case never happened.
  *[Reward and Punishment (R&P):]* In this treatment each player can choose to either reward or to sanction another co-player of the same group. Players can give both rewarding and sanctioning response to different co-players in the same round, but not to the same player.

The prize to be won, *z*, constitutes a sort of local public good, as its consumption is non-rival and non-excludable within the group. All group members receive it and no-one can be excluded.

Note that players do not get to know from whom they get response points assigned. So neither do they know in the punishment treatment, which of the co-players sanctioned them, nor do participants get revealed by whom they receive reward tokens in the reward setup (equivalent for the R&P treatment). This feature is equivalent to the setup in Sefton et al. [9]. Its purpose is to circumvent that players react on response behaviour of particular co-players—think hereby of retaliation or gift exchanging. Furthermore, participants do not get to know about the response other players received. It also needs to be pointed out, that by rewarding other group members, participants shift around tokens in a way that leaves total welfare unchanged. Punishing others, in contrast, reduces overall welfare.

We apply a cost for response-giving of one as in Sefton et al. [9]. This means if player *k* punishes player *l* by 6, *k* has to pay 6 for this action. Some studies incentivise response-giving by relatively cheapening it (cf. [6, 15]). Fehr & Gächter [5] apply a non-linear cost function, where higher punishments are more costly (Casari [29] coins the term “fine-to-fee” ratio and shows that a non-constant “fine-to-fee” ratio can be problematic). We opt for a constant cost of one in order to keep both the punishment and the reward leverage small to mitigate confounds from efficiency preferences. Also, in case we applied unequal leverage for the two types of response, the difference could be confounding as well.

### Equilibrium strategies

In this subsection we discuss various theoretical benchmarks to the game that is played in the experiment. It is not the purpose to give point predictions of behaviour but rather to give some insights about the comparative statics. These in turn help to formulate hypothesis. For details throughout this subsection we refer to the S1 Appendix “Mathematical Appendix”.

A natural benchmark to compare our data with is the sub-game perfect equilibrium of the single-shot game. We derive this by backward induction. In the second stage, costly response only reduces own payoff. Hence, individualistic players do not give any response. Given this second-stage behaviour, players do not take punishment or reward into account when making the optimal decision about how many lottery tickets to buy for their group. Each player *l* maximises her expected payoff *π*_*l*_ which is

$$\begin{array}{c} \pi_{l}\left(v\right)=\frac{v_{l}+\sum_{k\in K\backslash \{l\}}v_{k}}{v_{l}+\sum_{k\in K\backslash \{l\}}v_{k}+\sum_{m\in M}v_{m}}\cdot z-v_{l}. \end{array}$$ (3)

This is the [28] winning probability (as introduced in Eqs 1 and 2) times the prize minus the cost for investing in the group project. For treatments without opponent ∑_*m*∈*M*_ *v*_*m*_ is set to 25. In this type of contest game with homogeneous groups, first order conditions yield a unique equilibrium with respect to the aggregate group contribution. On the individual level, however, multiple equilibria exist [27, 30, 31].

For all treatments, the equilibrium investment level for group *K* is $\sum_{k\in K}v_{k}=\frac{z}{4}$. For *z* = 100, this results in a Nash-equilibrium of 25 tokens per group. One conceivable solution to the within-group “burden-sharing” would be to assume symmetry as in Katz et al. [27]. For *z* = 100 and |*K*| = 4 this would result in an individual contribution of *v*_*l*_ = 6.25 tokens. Of course, this is only one example among many possible solutions to the within-group problem. Note that only integer amounts of investment are allowed, so 6.25 can best be viewed as an approximation.

Unlike the single-shot Nash-equilibrium, the social optimal strategy differs somewhat between the cases with or without an opponent and whether one regards the opponent team as part of the social system. In the treatments without opponent, the prize is forfeited and goes to nobody if it is not won. In this environment expected total monetary welfare is maximised at ∑_*k*∈*K*_ *v*_*k*_ = 75 tokens per group. Things look differently, however, if two groups compete against each other. Now the optimal strategy to maximise total monetary welfare across groups would be for both groups to invest nothing and face a 50:50 chance of winning. The rationale for this is straightforward: First of all no reward or punishment will be distributed, as this is either welfare neutral (rewarding) or reducing welfare (punishing). Buying lottery tickets is unproductive effort that only influences winning probabilities. One of the two groups wins the prize anyway and regarding total welfare of both groups, it does not matter, which of the two wins it. These steps of analysis are true for all periods of the game. If the opponent group is not regarded as part of the social system, expected total monetary welfare of a given group is maximised by using team reasoning as in Sugden [32], i.e. each team maximises expected earnings. Under this situation, the solution would be ∑_*k*∈*K*_ *v*_*k*_ = 100 tokens for each team. A crucial side effect of our implementation is that while in the between group contest environment, the social optimum and the team reasoning equilibrium are very different (0 and 100 respectively), both are identical in the non-contest environment. Ideally, all benchmarks were identical across treatments but this is not feasible. Egas & Riedl [33] find that the use of punishment is strongly governed by its cost-to-impact ratio. We expect a similar effect in our study.

Being in a contest with another group creates a process of social categorisation and collective identity [34], which in turn contributes to a development of competition, driven by an intergroup bias. These dynamics (also called: in-group favouritism and out-group spite) can be described as an inclination to benefit players from the own group and harm those from the competing group [20]. We model this by a very simple other-regarding utility function, extending Eq 3, in a similar way as in [35]. Average payoffs of other players from the *own* group *K* and the *other* group *M* factor into a player’s utility, using the weights $\theta_{K}^{C}$ and $\theta_{M}^{C}$, respectively.

$$\begin{array}{c} u_{l}\left(v\right)=\pi_{l}\left(v\right)+\theta_{K}^{C}\cdot \frac{1}{3}\cdot \sum_{k\in K\backslash \{l\}}\pi_{k}\left(v\right)+\theta_{M}^{C}\cdot \frac{1}{4}\cdot \sum_{m\in M}\pi_{m}\left(v\right) \end{array}$$ (4)

For the non-contest environment we assume that players care about others weakly positively with weight $\theta_{K}^{N}$ as being found in the overwhelming majority of the literature (e.g. [36–38]). The part for the other group *M* collapses to zero. The resulting utility function is

$$\begin{array}{c} u_{l}\left(v\right)=\pi_{l}\left(v\right)+\theta_{K}^{N}\cdot \frac{1}{3}\cdot \sum_{k\in K\backslash \{l\}}\pi_{k}\left(v\right). \end{array}$$ (5)

To keep the model simple we assume identical parameters for each player. Further we assume that the parameters rank as discussed in the psychological literature above.

$$\begin{array}{c} 1>\theta_{K}^{C}>\theta_{K}^{N}\geq 0>\theta_{M}^{C}>-1 \end{array}$$ (6)

Solving the model in the contest environment leads to a multiplicity of Nash-equilibria, characterised by

$$\begin{array}{c} \sum_{k\in K}v_{k}=(1+\theta_{K}^{C}-\theta_{M}^{C})\cdot \frac{z}{4} \end{array}$$

and to a symmetric individual contribution of $v_{l}=(1+\theta_{K}^{C}-\theta_{M}^{C})\frac{z}{16}$. For the non-contest environment the Nash-equilibria are characterised by

$$\begin{array}{c} \sum_{k\in K}v_{k}=(2\sqrt{1+\theta_{K}^{N}}-1)\cdot \frac{z}{4} \end{array}$$

and the symmetric individual contribution is $v_{l}=(2\sqrt{1+\theta_{K}^{N}}-1)\cdot \frac{z}{16}$. The Nash-equilibria characterisation and Condition (6) lead to

**Hypothesis 1** *Contributions to the team contest are higher in the contest environment than in the non-contest*.

Our social preferences alone cannot explain that people punish or reward. However, in combination with reciprocal motives which are not explicitly modelled, this is feasible. Given all else equal, the payoff of other players from the own group factors more strongly into a player’s utility in the contest environment $\left(1+\theta_{K}^{C}-\theta_{M}^{C}\right)$ than in the non-contest environment $\left(1+\theta_{K}^{N}\right)$. Consequently, punishing becomes relatively more expensive, as the reduction in other teammates’ payoff through punishing translates into a reduction of own utility. In contrast, rewarding becomes cheaper because rewards for others feed back positively in ones own utility. We therefore state the following related hypotheses with respect to rewarding and punishment behaviour.

**Hypothesis 2** *In a team contest, rewarding is more extensively used than punishment*.

**Hypothesis 3** *Players use more rewarding in the contest environment than in the non-contest*.

**Hypothesis 4** *Players use less punishing in the contest environment than in the non-contest*.

### Procedures

We used the system ORSEE by Greiner [39] to recruit a total of 372 participants (most of them students of Business and Economics) for our experiment. Each participant received a financial compensation for taking part in the experiment. This compensation was dependent on the total amount of tokens earned over the fifteen rounds. The experiment took about one hour, including reading the instructions, a trial period, the contest game as such, a questionnaire and payment. The mean income was €16.94 across all treatments ($22.48 at the time of the experiment). While players in treatments without opponent received an average of €19.61, players in treatments with opponent received €15.60 on average.

The experiment was programmed in z-Tree [40] and conducted at the facilities of the BEElab (Behavioral & Experimental Economics Laboratory, Maastricht University, Netherlands). Each participant sat in her own cubicle, where she made her decisions, physically and visually separated from other participants. Upon entering the cubicle, each participant found the instructions at her place (see S1 Appendix “Instructions”). After reading the instructions, participants answered some trial questions, in order to become familiar with both the conceptual setup of the game, as well as with its user interface. When the experiment was finished, players were asked to fill out a short questionnaire about personal features to be used in the analysis of the game.

In the trial phase, players were confronted with randomly generated game situations and answered control questions. They were aware of the randomness of the numbers presented and also knew that they were not yet interacting with other participants. In general, participants got to see three different screens per period: First there was a screen where each player decided how much to contribute for the group account. Then the player got to know whether her group has won, own winning probability and the contributions of fellow group members. She then had the opportunity to assign response tokens to co-players. In the baseline treatment, this was just an overview page, without the opportunity to assign response tokens. On the third screen, the total profit (in tokens) for this period was displayed in a detailed overview for the participant (see S1 Appendix “Stages”).

## Results

We begin by comparing contribution levels and response over time. Then we examine, which effects drive the differences in behaviour at the individual level. We find that participants’ actions are heavily influenced by what happened in previous rounds. Therefore we take a closer look at the dynamics of this experiment in Subsection Dynamics in Decision Making. There is a strong relationship between contribution to the group account and response-giving. We analyse the relationship between contributions and receiving reward or punishment in Subsection Who receives Response?.

In this study, individual observations per period are not independently distributed, as the actions of other participants and previous rounds influence own behaviour. In line with this, contribution shows a high degree of autocorrelation and heteroscedasticity. This is why we use Newey-West standard errors for our regressions, as devised by Newey & West [41], with a lag length of two periods (Rule of thumb by Stock & Watson [42]: use lag length of 0.75⋅*T*^1/3^ with *T* being the number of rounds in the experiment). As alternative regression method, we run OLS regressions with standard errors that cope for heteroscedasticity and clustering between individuals, as described in Angrist [43, Chapter 8].

We use non-parametric methods to test the hypotheses: Mann-Whitney U tests (MWU) [44] for independent sample tests and Wilcoxon signed-rank test [45] for paired tests. While in the non-contest environment, group level data (four players) constitutes an independent observation, we use paired group data (eight players) in the contest environment. All tests are executed two-sided.

### Contribution to the group account

Average group level contribution over all treatments is at about 142, which is significantly higher than the Nash equilibrium as benchmark (Wilcoxon test: *N* = 62, *p* = 0.000). If analysed separately, contribution levels in both environments and in all treatments are significantly higher than the Nash equilibrium benchmark as well.

**Fig 1:**
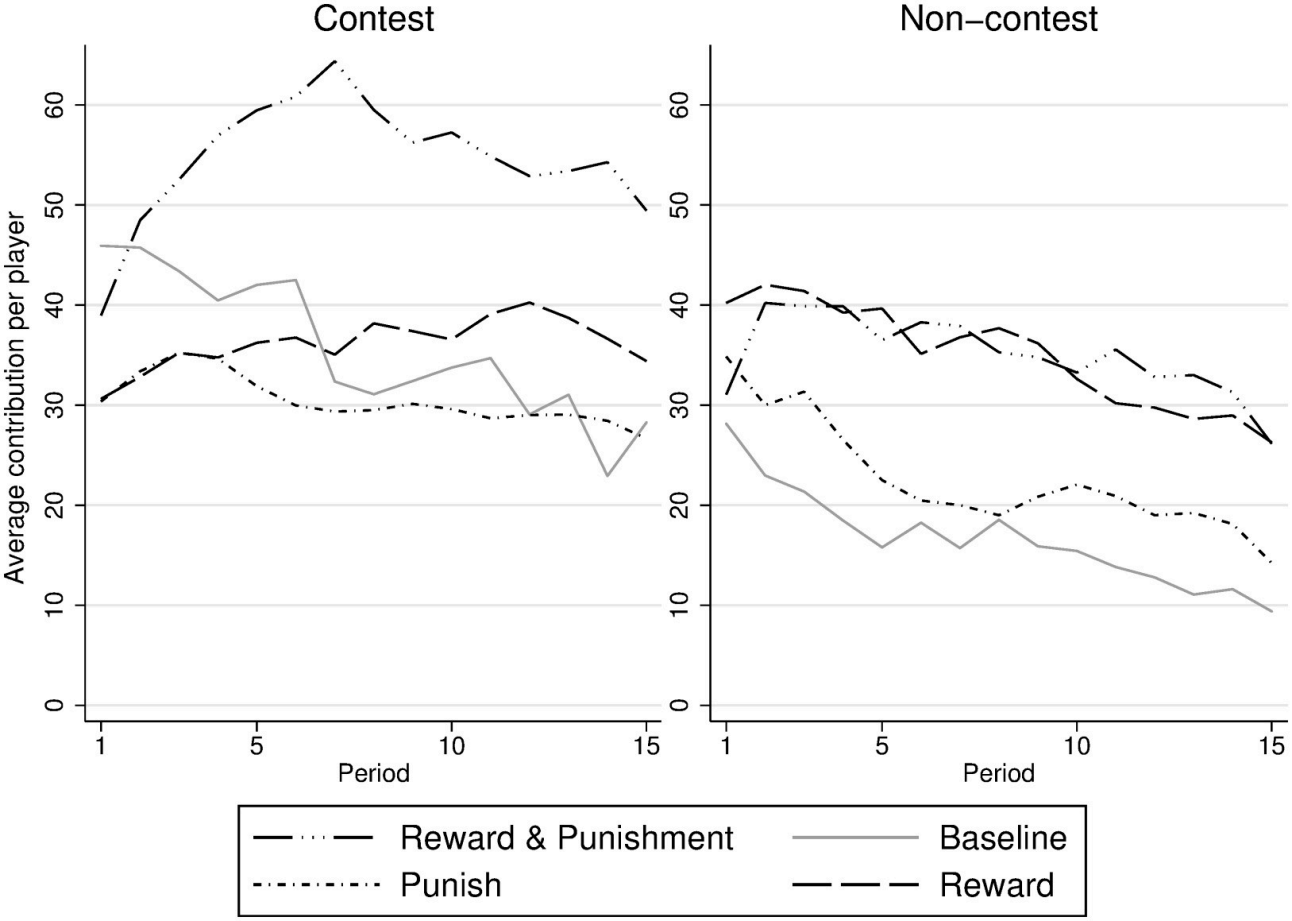
Individual contribution to the group account per treatment. Contest environment on the left, non-contest environment on the right.

Fig 1 illustrates average contribution to the group account per participant over all 15 rounds. Results for the contest environment are on the left and for the non-contest on the right. While contributions in the reward treatment for the non-contest environment are indeed higher than for the respective punishment treatment (MWU test: *N* = 16, *p* = 0.016), this is not the case for the contest environment (MWU test: *N* = 16, *p* = 0.529).

Most striking, however, is the result for the R&P treatment in the contest environment, which peaks out over all other ones (MWU test: *N* = 62, *p* = 0.001). Also, the comparably high contribution rate for the baseline treatment in the contest environment is remarkable. As such, unlike in Abbink et al. [15], allowing team members to punish each other does not lead to higher expenditures into the contest in our study (MWU test: *N* = 15, *p* = 0.133).

**Fig 2:**
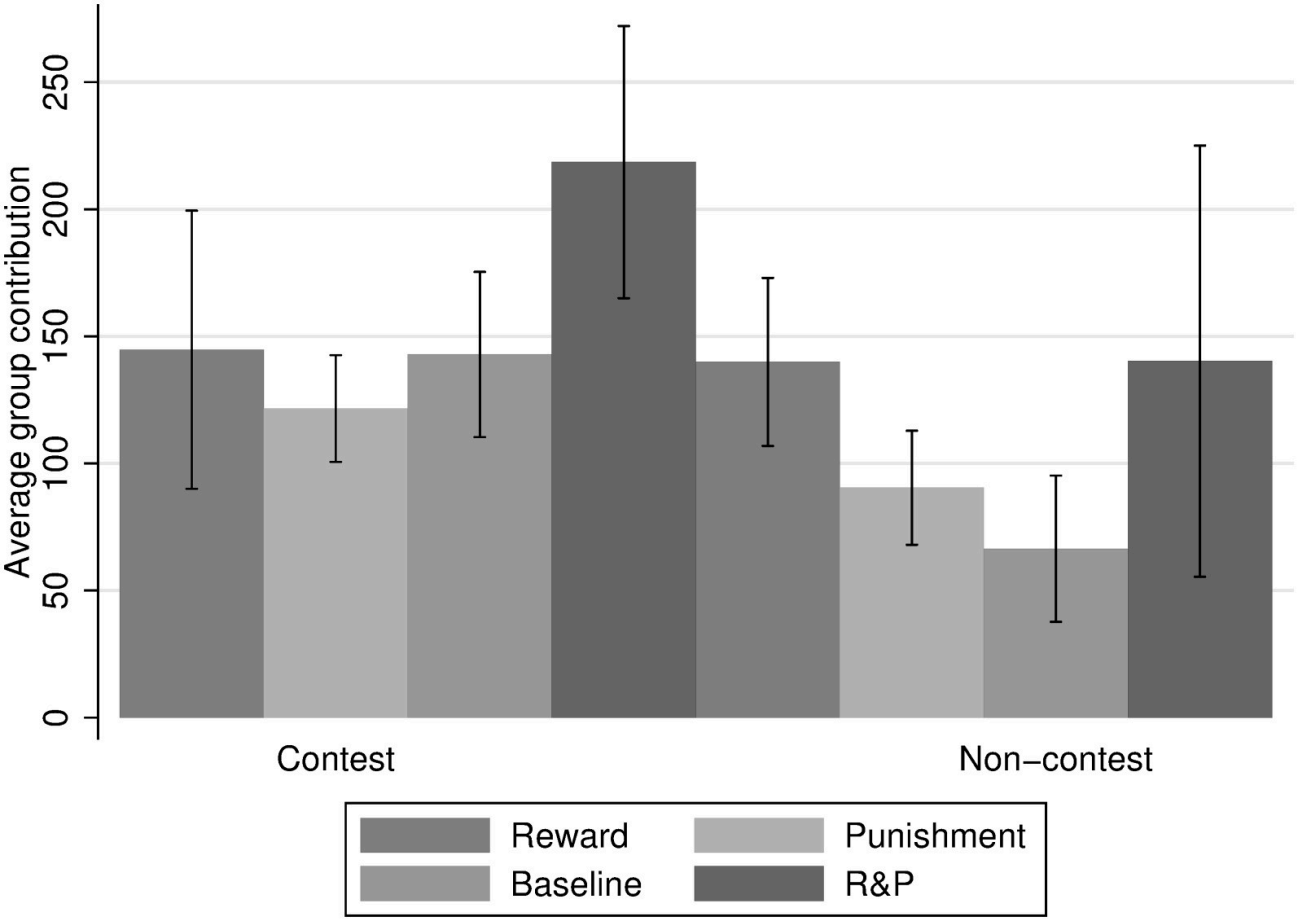
Average contribution to the group account per treatment. Contest environment on the left, non-contest environment on the right.

In Fig 2, average group contribution per treatment can be compared. The error bars give the 5% confidence interval, on the basis of independent observations, which is the average contribution per pair of groups over all 15 periods.

By and large, contribution levels averaged over all rounds are on a fairly similar level for the reward treatment in both environments. For all other treatments, contribution levels are lower in the non-contest environment, delivering some first support for Hypothesis 1. At the same time, though, even within the treatments, there exists considerable heterogeneity between groups’ contribution levels (see S1 Appendix “Group wise Analysis of Contribution”).

### Response giving

**Fig 3:**
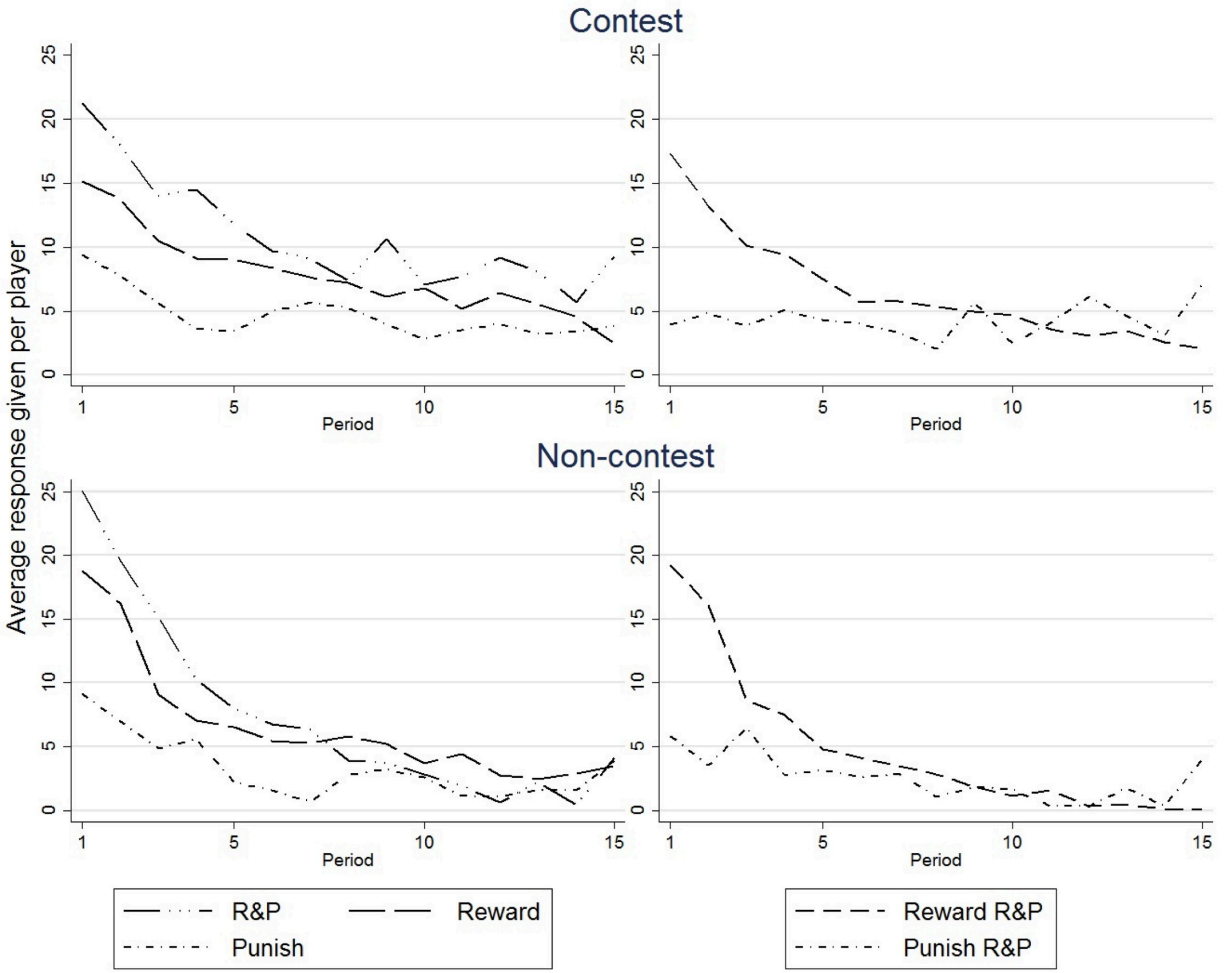
Response given per treatment.

As Fig 3 illustrates, a systematic difference for the response-giving, can be identified between the treatments, but also within the R&P treatment. As for Fig 1, we report mean values for each treatment and period per participant. The two graphs in the left column depict average spending levels for the treatments with response. On the right hand column, rewarding and punishing in the R&P treatment are compared. The first row is for the contest and the second row for the non-contest environment.

Comparing rewarding and punishing behaviour, it seems that the former is slightly more extensively used, especially in the beginning of the experiment. In the R&P treatments (analysing both environments together and separately), rewarding is significantly higher than punishing (e.g. Wilcoxon test including both environments: *N* = 16, *p* = 0.03). This relationship also holds between the reward and punishment treatments when both environments are analysed together (MWU test: *N* = 32, *p* = 0.029), but does not deliver a significant result for the separate environments. Both environments seem to not differ significantly in their propensity to use reward (MWU test: *N* = 32, *p* = 0.2) or punishment (MWU test: *N* = 32, *p* = 0.105).

Especially in the beginning of the game, reward giving is used more than punishing, while the two approach each other over time, even turning punishing into the preferred form of response giving for the last period in the R&P treatment (e.g. Wilcoxon test average reward versus average punishment in period 15 at group pair level including both environments: *N* = 16, *p* = 0.012). We conclude that participants tend to behave more favourable towards other group members in the beginning. In the course of the game, however, friendliness fades out.

**Fig 4:**
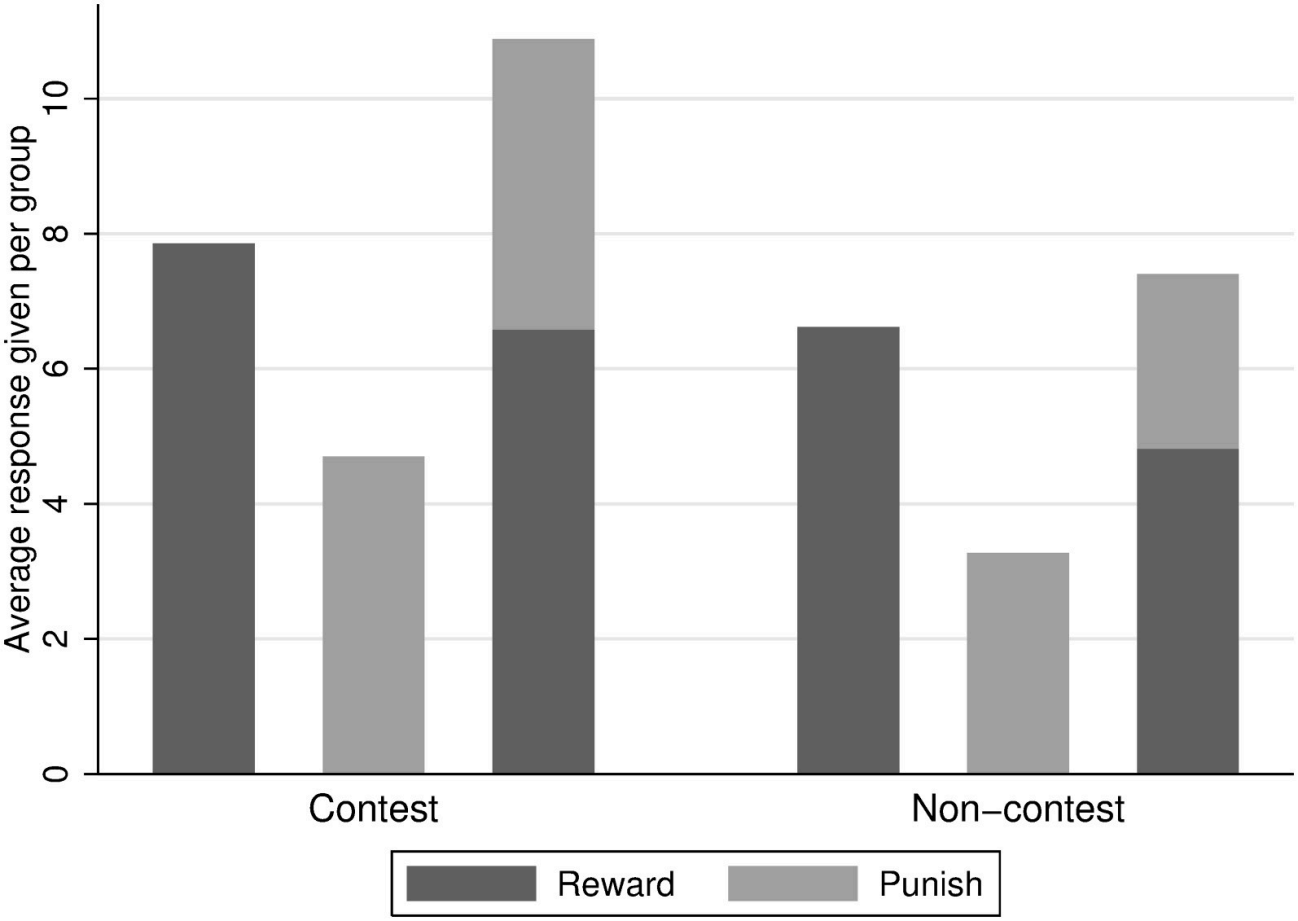
Response given per treatment.

Also, overall response giving is significantly higher in the R&P treatment than in the other treatments (MWU test: *N* = 48, *p* = 0.002). Especially in the contest environment there seems to exist a somewhat robust demand for rewarding or punishing. This means that participants seem to not have a common mental budget for overall response, but a separate budget for each rewarding and punishing. For this consider also Fig 4.

Tables 1 and 2 show the propensity to engage in rewarding and/or punishing behaviour for each environment. It shows the percentage of non-negative response per treatment and the type of response being sent. While the propensity to punish is fairly similar between the R&P and the punishment treatment in each environment (25 and 27% in contest environment, both 19% in non-contest environment), more participants engage in rewarding behaviour in the reward treatment, as compared to the R&P treatment (56% in reward contest environment, 57% in reward non-contest environment, compared with 44% and 26% respectively in R&P). Unlike in the R&P treatment, participants in the reward treatment can only reciprocate negatively by not sending rewarding tokens. The spectrum of reciprocal actions shifts accordingly. In the punishment treatment this shift does not happen.

**Table 1:** Share of response cases, contest environment (in percentages).

	Punish						
Reward	No	Yes	Total	Reward		Punish	
No	46	11	56	No	44	No	73
Yes	29	14	44	Yes	56	Yes	27
Total	75	25	100	Total	100	Total	100
R&P				Reward		Punish	

**Table 2:** Share of response cases, non-contest environment (in percentages).

	Punish						
Reward	No	Yes	Total	Reward		Punish	
No	62	11	74	No	43	No	81
Yes	18	8	26	Yes	57	Yes	19
Total	80	19	100	Total	100	Total	100
R&P				Reward		Punish	

Between the environments, notice the close resemblance of percentages in the reward treatment. Notice also an increase in response giving behaviour for the contest environment, except for the reward treatment. The results of this subsection deliver support for Hypothesis 2, but not for Hypotheses 3 or 4.

In Tables 3 and 4 we present results from OLS regressions per treatment with Newey-West standard errors regressing response-giving in a particular period on a binary win term (i.e. 0 if your group lost, 1 if it won) and a lagged response term as control. While winning has no impact on punishing behaviour, participants seem to increase rewarding others upon having won in three out of four regressions.

**Table 3:** Contest environment. OLS regression with Newey-West standard errors.

VARIABLES	(1)	(2)	(3)	(4)
	Reward	Punish	Reward R&P	Punish R&P
	Response given			
Own response Lag 1	0.537***	0.590***	0.329***	0.206***
	(0.04)	(0.05)	(0.04)	(0.04)
Win	2.128***	0.394	1.231*	−0.581
	(0.48)	(0.60)	(0.70)	(0.82)
Constant	8.564***	2.531*	2.215*	−0.283
	(1.76)	(1.53)	(1.34)	(1.58)

* p<0.05,

** p<0.01,

*** p<0.001

Standard errors in parentheses. Netherlands & Belgium, group and study major control variables not reported.

**Table 4:** Non-contest environment. OLS regression with Newey-West standard errors.

VARIABLES	(5)	(6)	(7)	(8)
	Reward	Punish	Reward R&P	Punish R&P
	Response given			
Own response Lag 1	0.350***	0.574***	0.466***	0.109***
	(0.07)	(0.09)	(0.05)	(0.04)
Win	2.365***	0.193	1.129	−0.178
	(0.71)	(0.73)	(0.79)	(0.91)
Constant	2.516**	0.191	−1.209	2.104
	(1.20)	(0.80)	(1.01)	(1.49)

* p<0.05,

** p<0.01,

*** p<0.001

Standard errors in parentheses. Netherlands & Belgium, group and study major control variables not reported.

### Rent dissipation

Deviations from equilibrium strategy (as devised in Subsection Equilibrium Strategies) of course have payoff-relevant consequences. As mentioned before, mean income was €16.94 taken across all treatments. This translates to an amount of 2,540 tokens per participant. If everyone exhibited equilibrium behaviour all throughout the experiment, everyone could have earned on expectation

$$\begin{array}{c} E\left(\pi_{tot}^{Nash}\right)=(B+p_{K}\cdot z-\frac{\sum_{k\in K}v_{k}}{4}+F)\cdot 15 \end{array}$$

or

$$\begin{array}{c} (100+0.5\cdot 100-6.25+50)\cdot 15=2,906.25 \end{array}$$

tokens, or €19.38, respectively. So overall, participants earn almost 15% less than what they could earn in Nash equilibrium.

In comparison, the socially optimal expected payoff amounts to $E(\pi_{tot}^{soc})=3,000$ tokens or €20.00 in the contest environment (with *v*_*i*_ = 0 ∀ *i* ∈ *K* ∪ *M*) and $E(\pi_{tot}^{soc})=2,718.75$ tokens or €18.13 in the non-contest environment (with *v*_*i*_ = 18.75 ∀ *i* ∈ *K* ∪ *M*).

Losses in total monetary welfare result from higher contributions, as compared to payoff-optimal strategies. This particularly emerges in the R&P treatment of the contest environment, where mean income was about €3.94 or 591 tokens (about 22%) lower than in the other treatments. Also, as a result of higher spending and punishing, average income in the contest environment was roughly €4.00 lower than in the non-contest (about 601 tokens, 20%).

Table 5 gives an overview on the extent and composition of overspending per treatment. We compare the total sum of individual contest expenditures and response giving with the Nash equilibrium benchmark and report the respective spending level that exceeds this threshold. The total level of overspending in each treatment of the non-contest environment is lower than its counterpart in the contest environment. For the Baseline treatments this decline is even as high as 60%. Column 3 delivers further support for Hypothesis 2. For all treatments, overspending on response tokens is higher in the contest environment. Column 3 further conveys evidence in favour of Hypothesis 3 (more rewarding in the contest environment), yet also evidence against Hypothesis 4 as participants spend more on punishing in the contest environment than they do in the non-contest.

**Table 5:** Individual overspending compared to the Nash equilibrium benchmark.

Treatment		Overspending contribution	Overspending response	Total
Contest	R&P	725.73	163.27	889.00
	Reward	448.98	117.84	566.83
	Punish	362.19	70.48	432.67
	Baseline	441.91	−.−	441.91
Non-contest	R&P	432.09	111.06	543.16
	Reward	431.03	99.25	530.28
	Punish	245.34	49.13	294.47
	Baseline	155.46	−.−	155.46

### Individual level analysis

We analyse, which factors influence individual behaviour in this game, using clustered OLS regression. For this, we include answers from the questionnaire that each participant filled in after the experiment (see S1 Appendix “Personal attributes” for an explorative analysis of additional control factors). Analogous to Subsection Response Giving, we drop the baseline treatment for conceptual reasons, as participants in this particular treatment cannot give a response. Table 6 presents the results. Regressions (9) and (10) show individual contribution, averaged over all 15 periods, on a number of factors. In (11) and (12) we regress own response on a similar set of factors.

**Table 6:** Individual level analysis. OLS regression with clustered standard errors at the group level.

VARIABLES	(9)	(10)	(11)	(12)
	Contribute	Contribute	Own response	Own response
Contribute			0.238***	0.268***
			(0.04)	(0.04)
Own response	0.714***	0.815***		
	(0.14)	(0.14)		
Group contribution level (excl. self)	0.282***	0.276***	−0.050***	−0.064***
	(0.01)	(0.02)	(0.01)	(0.01)
Group response level (excluding self)	−0.150***	−0.154**	0.143***	0.143***
	(0.05)	(0.06)	(0.04)	(0.05)
Other group contribute	0.008*	0.007	0.003	0.004
	(0.00)	(0.01)	(0.00)	(0.00)
Constant	2.911***	51.418***	0.479	−20.705**
	(0.92)	(12.88)	(0.57)	(8.14)
Controls	No	Yes	No	Yes
R-squared	0.698	0.749	0.306	0.426
N	288	270	288	270

* p<0.05,

** p<0.01,

*** p<0.001

Standard errors in parentheses.

As for the factors “Contribute” and “Own response” it can be seen that participants who give more response, tend to also be those who contribute more and vice versa, which is very intuitive: Those who display more involvement in the project, both contribute more and give more response in order to induce higher contribution by other group members as well.

Results for the average per-period sum of contributions of group members excluding oneself (“Group contribution level (excluding self)”) indicate that participants in a competitive group join in and spend more resources for the contest themselves. At the same time, response giving is slightly reduced. For the average per-period sum of response of group members excluding self (“Group response level (excluding self)”), roughly the opposite relationship applies. We will examine the effect of response on individual behaviour more closely in Subsection Dynamics in Decision Making. Individuals might not only be affected by their own group’s actions, but also by those participants across the aisle. To this end, “Other group contribute” is the sum of contributions of the opposing group, averaged over the 15 periods. This seems to only marginally affect individual contribution decisions and shows only some degree of statistical significance in Regression (9).

### Dynamics in decision making

For this part, we closely follow the analysis technique of Ashley et al. [46] and Sefton et al. [9]. We employ OLS regression per treatment with Newey-West standard errors using the contribution to the group account as dependent variable. Tobit regressions lead to similar results. The explanatory variables are the contribution to the group account lagged for one and two periods, individual positive or negative deviation from other group members’ contribution level lagged one period, and response received one period ago. We analyse the effect of receiving reward or punishment, respectively, on subsequent contribution levels. Results are presented in Tables 7 and 8.

**Table 7:** Contest environment: Dynamic analysis. OLS regression with Newey-West standard errors.

	(13)	(14)	(15)	(16)
	Reward	Punish	Baseline	R&P
VARIABLES	Contribute			
Contribution to group account in previous period	0.440***	0.573***	0.297**	0.634***
	(0.09)	(0.08)	(0.09)	(0.08)
Contribution to group account lagged two periods	0.264***	0.169**	0.223***	0.252***
	(0.05)	(0.05)	(0.06)	(0.05)
Positive deviation from other group members in previous period	−0.257**	−0.116	−0.050	−0.332***
	(0.10)	(0.12)	(0.11)	(0.08)
Negative deviation from other group members in previous period	−0.172*	0.045	−0.111	0.178
	(0.08)	(0.06)	(0.07)	(0.10)
Reward received previous period	0.226*			0.216**
	(0.09)			(0.08)
Punishment received previous period		0.026		0.115
		(0.04)		(0.07)
Constant	24.930***	12.908**	28.556***	8.326*
	(1.94)	(4.62)	(5.33)	(3.90)

* p<0.05,

** p<0.01,

*** p<0.001

Standard errors in parentheses. Netherlands & Belgium, group and study major control variables not reported.

**Table 8:** Non-contest environment: Dynamic analysis. OLS regression with Newey-West standard errors.

	(17)	(18)	(19)	(20)
	Reward	Punish	Baseline	R&P
VARIABLES	Contribute			
Contribution to group account in previous period	0.611***	0.601***	0.363***	0.315*
	(0.09)	(0.10)	(0.10)	(0.14)
Contribution to group account lagged two periods	0.145*	0.120*	0.235***	0.235**
	(0.06)	(0.05)	(0.07)	(0.09)
Positive deviation from other group members in previous period	−0.059	−0.034	−0.037	−0.044
	(0.11)	(0.16)	(0.15)	(0.17)
Negative deviation from other group members in previous period	0.255**	0.083	0.064	0.383**
	(0.09)	(0.10)	(0.13)	(0.13)
Reward received previous period	−0.082			0.229*
	(0.11)			(0.09)
Punishment received previous period		−0.010		0.026
		(0.04)		(0.09)
Constant	7.851	9.268**	10.911**	41.306***
	(4.48)	(3.03)	(3.70)	(9.68)

* p<0.05,

** p<0.01,

*** p<0.001

Standard errors in parentheses. Netherlands & Belgium, group and study major control variables not reported.

Next to a significant autocorrelation for contribution levels, this analysis delivers two main insights: *First of all*, the lagged relative comparison of own contribution to the mean group contribution of the other three group members reveals that there seems to be a slight tendency for students contributing relatively more to the project to reduce their spending for the group account in the subsequent period. This turns out to be of significant effect only for Regressions (13) and (16), however. For the opposite relationship, six of the eight regressions point into the direction of increasing contributions in the period subsequent to being found contributing less than the team mates. Regressions (17) and (20) display significantly positive covariates in this regard. For Regression (13), however, this regressor is even significantly negative. This regression to the mean effect of reducing contributions when having contributed more before and increasing contributions when having spent less is not very strong in this game.

*Second*, in line with Sefton et al. [9], receiving rewards induces an increase in contribution for the following round in three of the four treatments, where it is available. Sanctions, on the other hand result in no significant change in behaviour on the receptor’s part. Sefton et al. [9] argue that this explains the negative trend for punishment-giving over the periods. Furthermore, they mention that reward-giving also declines in the course of the experiment, but at a faster pace than punishment-giving. This is the case despite reward inducing a stronger reaction on the part of other participants’ behaviour. It may seem odd that participants do not stick to a strategy that seems to be effective in inducing other participants to contribute. Yet given the size of the coefficient (i.e. 0.226, 0.216 etc.), it would be more efficient to simply increase own investment instead.

Fig 2 shows that the R&P treatment, in which participants can choose between reward and punishment, is substantially more effective in triggering high contribution to the contest than any other treatment. Furthermore, Fig 4 suggests that part of this effect is due to higher overall response in the R&P treatment. An alternative explanation would be that receiving reward or punishment in the R&P treatment is perceived differently. Participants might dislike receiving punishment more when the prospect of reward has been on the table. Our results do not lend support for this latter hypothesis, though. Coefficients for both “Reward received previous period” and “Punishment received previous period” in Regression (16) are very similar to their counterparts from Regressions (13) and (14).

We observe a similar regression to the mean effect, as reported in this subsection, when considering the effect of winning on contribution levels. In specific, having won in the previous period (*t* − 1), seems to reduce contribution levels in the subsequent period *t* only for groups with a relatively favourable winning probability (S14 Table in S1 Appendix “The Effect of Winning”).

### Who receives response?

In The Theory of Moral Sentiments, Adam Smith suggests: “Actions of a beneficent tendency, which proceed from proper motives, seem alone to require reward; because such alone are the approved objects of gratitude, or excite the sympathetic gratitude of the spectator. Actions of a hurtful tendency, which proceed from improper motives, seem alone to deserve punishment; because such alone are the approved objects of resentment, or excite the sympathetic resentment of the spectator” [47, Part II, Section II, Chapter I, Paragraph 1-2]. This early characterisation of reciprocity nicely captures our hypothesis concerning who will be punished or rewarded in this game.

For the contest environment, simple OLS regressions with Newey-West standard errors indicate that in the punishment treatment, participants who contribute less are more likely to receive punishment from their teammates. The opposite holds for the reward treatment. Participants who contribute more are more likely to receive reward from their teammates (see Tables 9 and 10). This relationship is true for both environments.

**Table 9:** Contest environment. OLS regression with Newey-West standard errors.

	(21)	(22)	(23)	(24)
	Reward	Punish	Reward R&P	Punish R&P
VARIABLES	Response received			
Contribute	0.260***	−0.636***	0.035	−0.269***
	(0.05)	(0.08)	(0.05)	(0.10)
Squared Contribute	−0.000	0.005***	0.000	0.001*
	(0.00)	(0.00)	(0.00)	(0.00)
Constant	−4.919***	15.428***	−0.197	16.739***
	(1.67)	(3.11)	(2.06)	(3.24)

* p<0.05,

** p<0.01,

*** p<0.001

Standard errors in parentheses. NLB dummy, group and study major fixed effects not reported.

**Table 10:** Non-contest environment. OLS regression with Newey-West standard errors.

	(25)	(26)	(27)	(28)
	Reward	Punish	Reward R&P	Punish R&P
VARIABLES	Response received			
Contribute	0.185**	−0.531***	0.372***	−0.107
	(0.08)	(0.14)	(0.09)	(0.10)
Squared Contribute	0.001	0.004***	−0.003**	−0.001
	(0.00)	(0.00)	(0.00)	(0.00)
Constant	−2.461	9.500**	−6.221	6.570
	(1.81)	(4.36)	(5.41)	(8.51)

* p<0.05,

** p<0.01,

*** p<0.001

Standard errors in parentheses. NLB dummy, group and study major fixed effects not reported.

When considering all periods, reverse causality cannot be ruled out: Perhaps participants contribute more if they are in a group in which more rewards are used (Regressions in Tables 7 and 8 present evidence for this) and contribute less in groups that tend to punish. In the S1 Appendix “Response received” we present results for Regressions (21)—(28), employing only data from period 1. All contribution decisions in the first period have been made absent of any prior response received. We find that the dichotomous reward/punishing relationship, as presented above, already exists in the first period.

**Fig 5:**
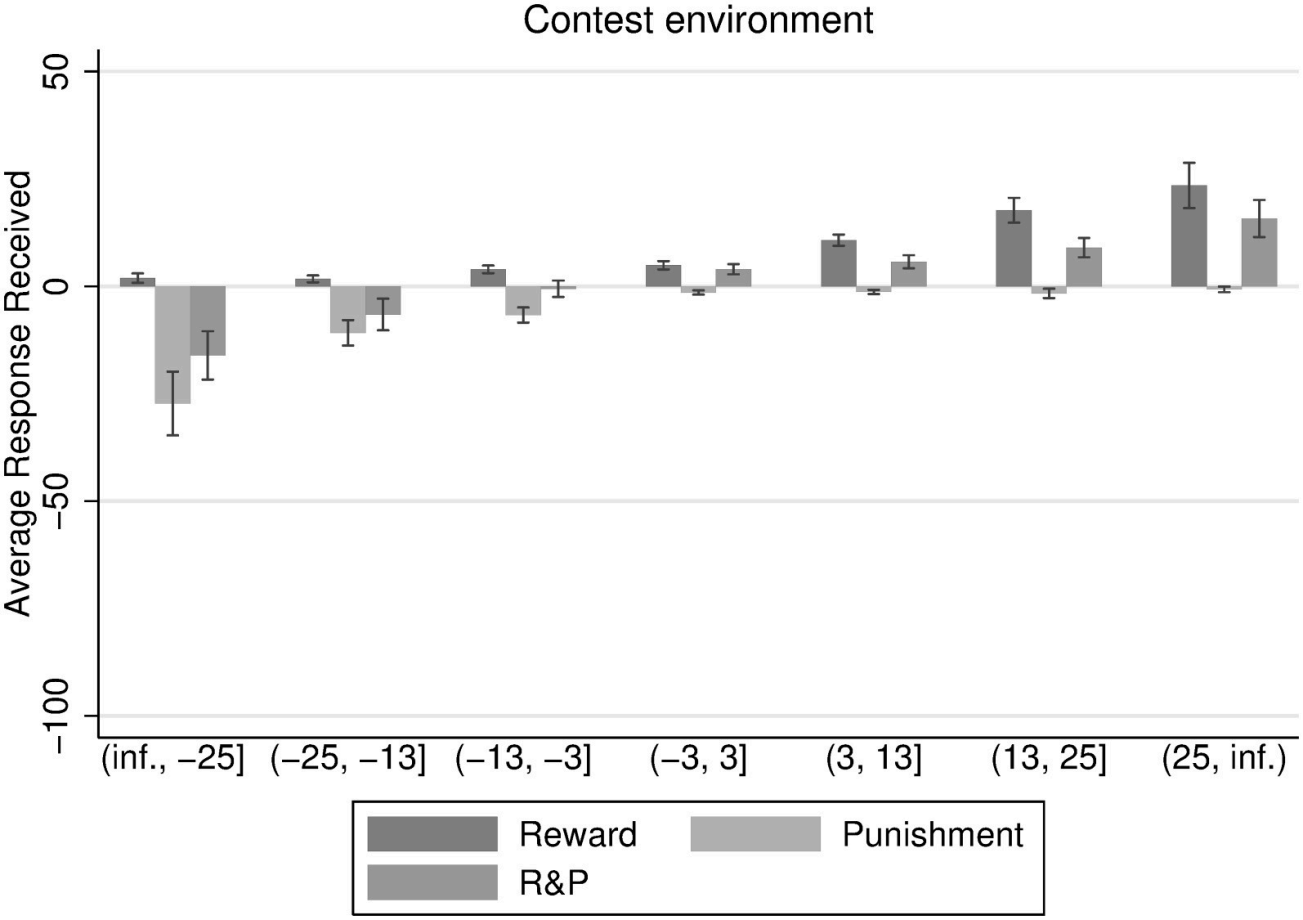
Response received in relation to deviation from average group contribution with 5% confidence interval.

Fig 5 displays response received as a function of individual’s deviation from the mean of contributions per group in the contest environment. These deviations are grouped into intervals, illustrating the gradient nature of response-giving. The higher the own deviation from the mean towards the positive region, the more reward and less punishment does a participant receive. We observe the contrary for punishment. The higher the deviations from the group’s mean contribution towards the negative array, the more punishment and less reward does a participant receive. We observe a small amount of asocial punishment, as well as some reward for participants that contribute less than the mean of the group contribution level.

Fig 5 suggests that the response methods are well in place, such that they are used to punish defectors and reward high contributors. Furthermore, the gradient of response-giving seems to be rather smooth. There seems to be an exponential relation between contributing and receiving response, such that participants who contribute much more than the mean of the group contribution, receive disproportionate reward. The same holds for the punishment treatment. Participants can expect to get disproportionately punished if they contribute much less than their fellow group members.

**Fig 6:**
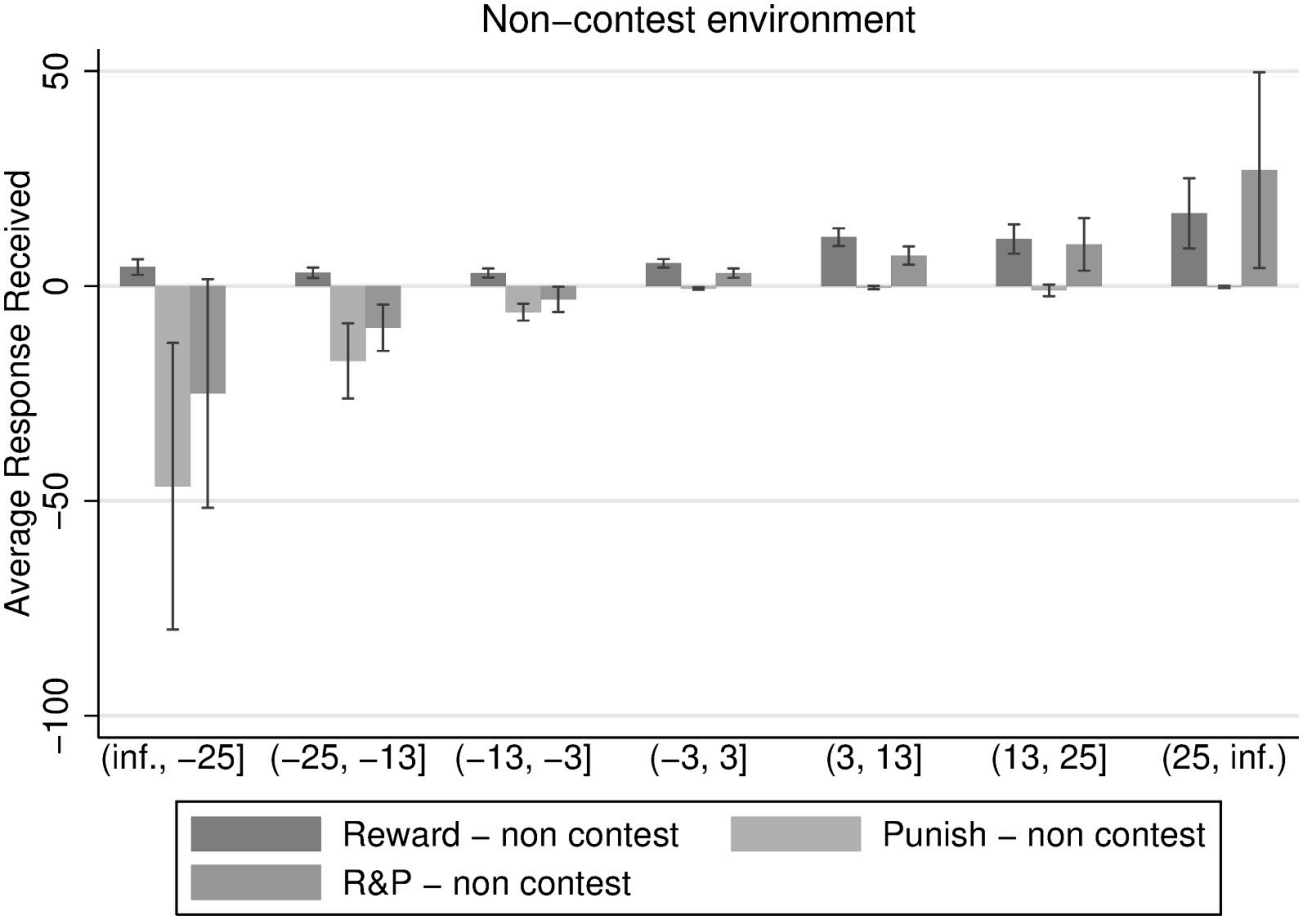
Response received in relation to deviation from average group contribution with 5% confidence interval.

We observe a similar, gradual composition of response received over deviations from average group contributions in the non-contest environment in Fig 6. Although the data is considerably more noisy than for the contest environment, a definite trend can be observed towards higher punishment of group-mates whose contribution deviates negatively from the group average, as compared to the contest environment. At the same time, this is not true for the positive domain: positive contributions are not rewarded more than in the contest environment.

Alternatively, individuals might actually compare others’ contribution levels to their own, when making their decision of whom to reward or to punish. Hence, it must not necessarily be the case that the mean of group contribution delivers the relevant benchmark. To capture this, we use a dyadic relation, instead of an average group contribution level. This means that clusters for relative contribution are not formed by comparing own contribution level to the group mean, but by comparing it to the contribution of the “response giver”. Hence, the deviation of own contribution to the contribution of those who punish or reward. This relative contribution, $dev_{l}^{k}=v_{l}-v_{k},$ gives a term of relative contribution for each of the three co-players in the group for a given period *t*. $dev_{l}^{k}$ measures the difference between contribution between player *l* and player *k*. Consequently, we get three $dev_{l}^{k}$-terms per player.

Results depicted in S14 and S15 Figs (in S1 Appendix “Response received”) stay qualitatively similar. They additionally show, however, that reward and punishment mechanisms are used in a redistributive way. Participants tend to recompense team mates who contribute more to the contest by means of reward and reduce the payoff of lower contributors (those who earn more in the contest) by punishing. Comparing S14 and S15 Figs in S1 Appendix also suggests that low contributors are punished more heavily in the non-contest environment.

## Discussion and conclusion

In this article we complement related work by Sefton et al. [9], Abbink et al. [15] and Fehr & Gächter [5]. We analyse a complex game of a team contest which entails both, aspects of a dilemma and aspects of a contest. We were particularly interested whether we find similar patterns of rewarding and punishment compared to the literature in public good experiments. We see this paper as building block towards a better general understanding on how institutional mechanisms like punishment and rewarding influence behaviour. Extrapolating from findings in public goods experiments does not seem to always work well. We consider Nikiforakis [48] a prominent example. He finds that the efficiency gains generated by the introduction of a punishment mechanism in a public good experiment are nullified by the introduction of a seemingly innocent possibility to counter-punish. Therefore we deem it necessary to build up a larger base of stylised facts that go beyond simple settings which prevail in the current literature. This enables the development of more robust theories on the nature of rewarding and sanctioning.

We contrast two treatments, one with contest and one without. We use a simple model of social preferences to derive some hypotheses: *1*) Contributions to the team contest are higher in the contest environment. *2*) In a team contest, rewarding is more extensively used than punishment. *3*) More rewarding in the contest environment as compared to the non-contest. *4*) Less punishing in the contest environment as compared to the non-contest.

Our results support Hypothesis 1 and provide plausible evidence in favour of Hypothesis 2. Hypotheses 3 and Hypothesis 4, however cannot be supported by our study. Next to the hypotheses tests we find some results in an explorative manner.

With respect to efficiency, the results of our experiment paint a gloomy picture. When confronted with a rival group, participants show a clear tendency to exhibit a more competitive behaviour. The opportunity to reciprocate teammates’ actions (by the means of reward or punishment), is utilised to fan the flames of the intergroup conflict, even though this comes at a price. Our results contribute to explaining the high humanitarian and material costs in socio-political conflicts. When confronted with a rival party, players seem willing to accept a materially inefficient outcome to outrun the opponent. All the more so, they incite their comrades to join in arms. In the anonymous and abstract environment of this computer laboratory, we find these outcomes absent of any religious or ethnic spur. In field environments, the emotional impetus attached to these kind of conflicts will be even stronger, given a usually higher social identity within the conflict parties.

We would like to note that our efficiency considerations do not always take all parties into account. In some of our motivating examples in the introduction, competition is not necessarily wasteful. Higher degrees of competition between architecture offices go along with higher values for the client and competitive matches between sports teams may create a huge value for spectators. It is therefore premature to derive policy recommendations that aim at reducing competition. In this respect it is interesting to see that in wage bargaining, measures have developed (conciliation, temporary peace obligations) to prevent wasteful competition (strikes and lock-outs).

In the following we neglect the question of efficiency and more narrowly focus on questions such as how rewarding and punishment are used and how they influence contribution behaviour. We find that across both environments, contribution to the group project is higher in the reward treatment than in the punishment treatment. Moreover, during the first rounds of the game, participants distribute more rewarding than punishing response to their group mates. We also find that for response-giving, the own contribution in relation to the contribution of other group members is an important determinant of the severity of punishment, or reward, respectively. This means that in the punishment treatment, the further a player’s contribution to the group account is below the mean of the group’s contribution level, the higher punishment this player can expect to receive from fellow group members. The opposite holds for the reward treatment. Here, the higher the relative contribution, the higher the expected reward from other group mates. Also, we find two dynamic patterns of behaviour: Players who contribute more than their group mates reduce contribution in subsequent periods, while the opposite does not hold. Players who contribute comparably less do not increase their contribution significantly. Most strikingly, however, players react on being rewarded by increasing their contribution, while players who get punished do not change their contribution level. Summing up we conclude that in our experiment the carrot works better than the stick. Unexpectedly it does so not only in the contest environment but also in the non-contest environment.

A few limitations apply to our experimental design. *First*, we cannot rule out that parts of our results are driven by a level effect, emanating from the design choice of employing a fixed amount of 25 blank tickets. This number is fairly close to the lower boundary of players’ decision space, which means that in terms of endowment, participants in our non-contest environment are comparably wealthy. Shifting the Nash equilibrium to higher contribution levels entails an increase of the prize. According to our model with other-regarding preferences this leads to higher contributions in the non-contest, but also in the contest environment. Recent studies have investigated this simple linear relationship. In a between group Tullock contest experiment, Baik et al. [49] investigate the effect of contest budget on contribution levels. They find a non-monotonic relationship such that contribution increases from low to medium budget, yet decreases from medium to high budget. Similarly, Schroyen and Nicolas [50] develop a model using a concave utility function with risk aversion to study wealth effects in a contest. They describe two opposing effects: wealth reduces the marginal cost of effort, yet decreases the marginal benefit of winning the contest. Schroyen and Nicolas [50] show that the final result from these two effects is ambiguous. In a field study, Miguel et al. [51] find evidence for raised levels of conflict in sub-Saharan Africa after a negative income shock (here: lack of rainfall), a result which is put into question by [52]. What these studies show is that the effect on contribution levels from raising the Nash equilibrium, and thus the amount of blank tickets is non-trivial and unclear. Eventually it remains an empirical question.

A *second* caveat may be the comparison between an environment with a dynamic opponent and one with a static amount of blank tickets. This was a deliberate design choice to avoid dynamics which emanate from the strategic interaction between the groups. Future studies may focus on investigating the relationship between static and dynamic non-human opposition. A *third* limitation may derive from the different efficiency implications of rewarding and punishing, respectively. While punishing other players reduces overall social welfare in the game (both distributing and receiving punishing tokens is costly), rewarding other players is an overall welfare-neutral activity (while distributing rewarding tokens is costly, being rewarded increases overall social welfare by the same amount). We try to keep this leverage as small as possible by applying cost for response-giving of one as in Sefton et al. [9].

There are a number of conceivable extensions: Allowing for communication could formalise a sort of non-binding contract between the participants (as in i.e. [53, 54]). In an experimental study using a group contest game with a weakest-link production function (total group contribution is determined by the lowest individual effort in that group), Cason et al. [53] allow for inter- and intra-group communication via a free form chat in a 2 × 2 design. They show that communication between groups has an abating effect on contest spending, which positively influences overall welfare. Within-group communication, by contrast, displays the opposite effect, leading to an escalation of the contest with a more than doubling of contest expenditures. In a treatment with both communication types, the two effects cancel out, leading to a level of competition and payoffs being not significantly different from the baseline treatment. Depending on the communication type this could potentially complement or counteract the rewarding and punishment effect.

By randomising identification numbers of players, reputation building would be excluded. Hypothetically, this should lead to a decrease in response-giving and also to a reduction in contribution to the group account. Going one step further, groups could even be set up in a randomised way. Group cohesion is expected to be even smaller than in the previous case, resulting in less response-giving and lower contribution levels.

In our design of the contest game, the prize for winning a round is set such that the Nash equilibrium for contribution is on a rather low level of players’ endowment with tokens. Throughout the game we constantly observe a massive over-investment across treatments. By varying the prize at stake, which changes the game’s Nash equilibrium, one could elicit the elasticity towards the contest prize. This might also have an effect on response-giving, making players react more aggressively towards non-contributors, with an increase in rewarding and punishing.

Summing up our study provides further evidence against the simplifying claim: “sufficient possibilities for peer-punishment will solve dilemmas”. To get a better picture on the nature of rewarding and punishment, even more stylised facts from even more complex situations have to be collected.

## Supporting information

S1 Appendix [55, 56]. (PDF)
