# Supplementary material for: Reward and punishment in a team contest
Source: PLoS One. 2020 Sep 17;15(9):e0236544. doi: 10.1371/journal.pone.0236544 (PMC7498014; doi:10.1371/journal.pone.0236544)
Supplement: S1 Appendix — (PDF) [file pone.0236544.s001.pdf]

Instructions

When entering the cubicle, each participant found a printed version of the following experiment instructions at her seat. Paragraphs headed by a treatment name in square brackets were only given to the participants of the corresponding treatment. The following example is from the contest treatments. Instructions for the non-contest treatments are slightly adjusted such as to not mention another group and avoid referring to teams or team mates. (Details available upon request).

CG Experiment

Welcome and thank you for participating in this experiment. Please read these instructions carefully. If you have any questions, please raise your hand and one of the hosts of the experiment will come to your cubicle to answer your question. Talking or using mobile phones or any other electronic devices is strictly prohibited. Mobile phones and other electronic devices should be left in the waiting room or switched off. If you are found violating these rules, you will both forfeit any earnings from this experiment, and may be excluded from future experiments as well.

This is an experiment about decision making. The instructions are simple and if you follow them carefully you might earn a considerable amount of money which will be paid to you privately and in cash at the end of today's session. The amount of money you earn depends on your decisions, on other participants' decisions and on random events. You will never be asked to reveal your identity to anyone during the course of the experiment. Your name will never be associated with any of your decisions. In order to keep your decisions private, do not reveal your choices to any other participant.

During the experiment you will have the chance to earn points, which will be converted into cash at the end of today's session, using an exchange rate of

$$15 \text{ point} = 0{,}1 \text{ €}.$$

Thus, the more points you earn, the more cash you will receive at the end of the session.

You and three other participants are assigned to a team. Four other participants are assigned to another team. All participants will remain in their teams for the entire experiment. None of you will learn who the own team members or the other team members are.

The experiment will consist of 15 periods, and in each period your team and the other team are competing for a prize in the following way:

At the beginning of each period you will receive 100 points. Then you can use these points to buy lottery tickets for your team. Any point you invest gives one lottery ticket for the team. Any point you do not invest in lottery tickets will be accumulated in your private point balance. Likewise, your team members can buy tickets for your team and the members of the other team can buy tickets for their team in exactly the same way.

As soon as everybody has chosen how many tickets to buy, a lottery will determine whether your team or the other team wins a prize of 400 points (100 for each team member). One of the sold tickets is randomly assigned the winning ticket. Each ticket has the same chance. Hence, the more tickets your team buys, the higher is your chance of winning the prize.

Examples: If your team and the other team buy the same amount of tickets then the chance of winning the prize is 50:50. If your team buys three times as many tickets as the other team, then also your team's chances are three times as high as those of the other team. If only one of the teams buys tickets then this team wins the prize with certainty.

If neither your team nor the other team buys a ticket, then the prize is randomly allocated to one of the teams with equal chances.

After the winning team is determined the prize of 400 points is equally shared and added to the private point balances of the winning team (100 points for each team member).

[Reward treatment:] At the end you get 50 feedback points. You can put these points to your private point balance or you can assign them to one or more of your team members (**not** to members of the other team). Each feedback point assigned to a team member increases this team member's private balance by one point.

[Punishment treatment:] At the end you get 50 feedback points. You can put them to your private point balance or you can assign the feedback points to one or more of your team members (**not** to members of the other team). Each feedback point assigned to a team member decreases this team member's private balance by one point. A team member's balance for one particular round cannot turn negative. If this happens then the balance for this particular round is set to zero and all excess feedback points are invalidated.

[Baseline treatment:] At the end you get 50 extra points which will add to your account.

[R&P treatment:] At the end you get 50 feedback points. You can put them to your private point balance or you can assign the feedback points to one or more of your team members (**not** to members of the other team). You can decide whether you assign the points to be added to the respective team member's account or to be subtracted from that account. Each feedback point assigned to a team member increases or decreases this team member's private balance by one point—depending on what you choose. A team member's balance for one particular round cannot turn negative. If this happens then the balance for this particular round is set to zero and all excess feedback points are invalidated.

The points you earn in each period will be added together. At the end of the session you will be paid based on your total point earning from all 15 periods.

The experiment starts with a trial period in which you will be asked to fill in some questions in order to check your understanding of the experiment and to give you the opportunity to get acquainted with the setup. Points earned in this trial period will not be paid off.

Stages

S1 to S3 Figs show screenshots from the punishment treatment. Other treatments look similar, adjusting only references to the payoff consequences of receiving response and leaving out mentioning of another group in the treatments without competing group.

Mathematical appendix

The game

There are two groups of players. Denote the in-group with K and a player from K with k. Similarly, denote the out-group and one of its players with M and m, respectively. Each player $i \in K \cup M$ invests an amount v_i for lottery tickets and receives an expected payoff of π_i. Denote the vector of investments with v, i.e. $v = (v_i)_{i \in K \cup M}$. For the non-contest environment group M

S1 Fig. First stage.

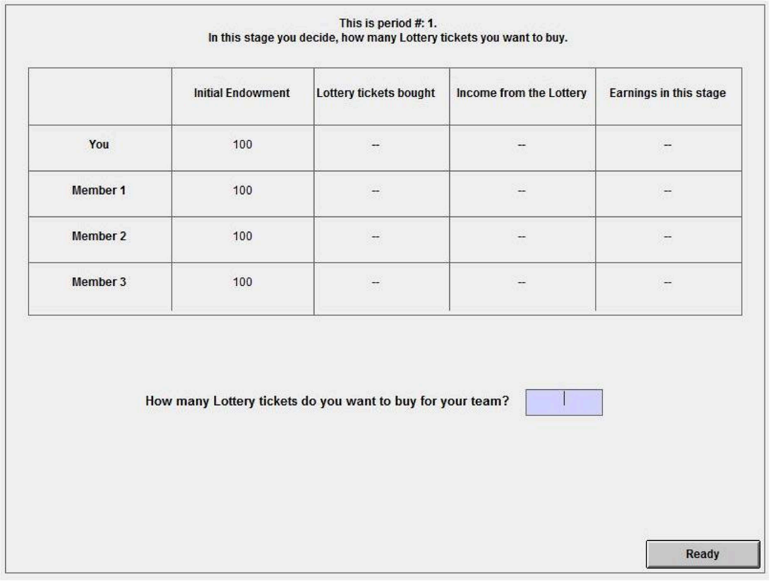

This is period #: 1.
In this stage you decide, how many Lottery tickets you want to buy.

	Initial Endowment	Lottery tickets bought	Income from the Lottery	Earnings in this stage
You	100	--	--	--
Member 1	100	--	--	--
Member 2	100	--	--	--
Member 3	100	--	--	--

How many Lottery tickets do you want to buy for your team?

Ready

S2 Fig. Second stage.

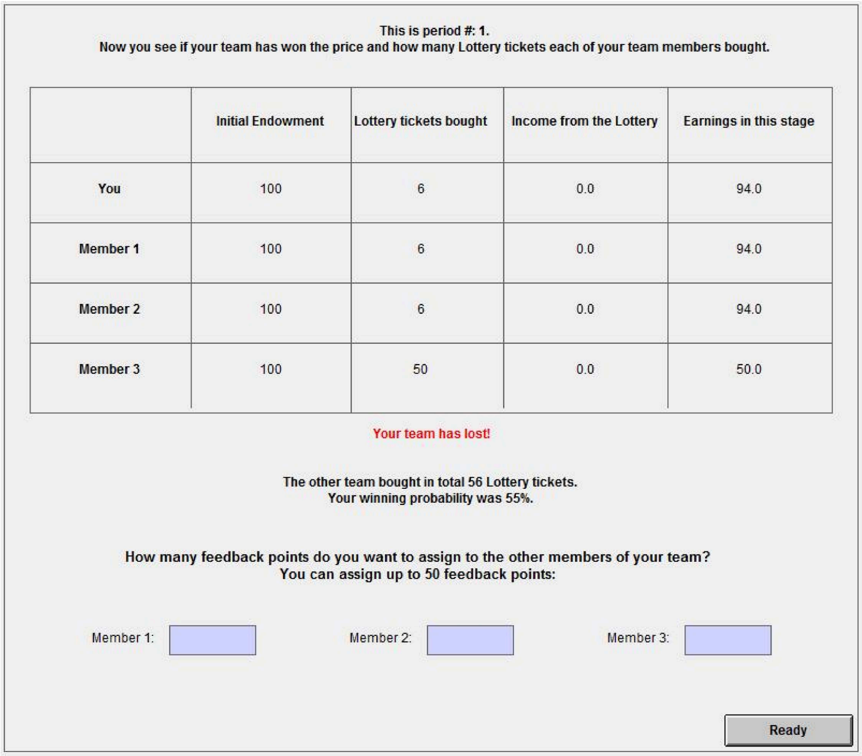

This is period #: 1.
Now you see if your team has won the price and how many Lottery tickets each of your team members bought.

	Initial Endowment	Lottery tickets bought	Income from the Lottery	Earnings in this stage
You	100	6	0.0	94.0
Member 1	100	6	0.0	94.0
Member 2	100	6	0.0	94.0
Member 3	100	50	0.0	50.0

Your team has lost!

The other team bought in total 56 Lottery tickets.
Your winning probability was 55%.

How many feedback points do you want to assign to the other members of your team?
You can assign up to 50 feedback points:

Member 1: Member 2: Member 3:

Ready

S3 Fig. Third stage.

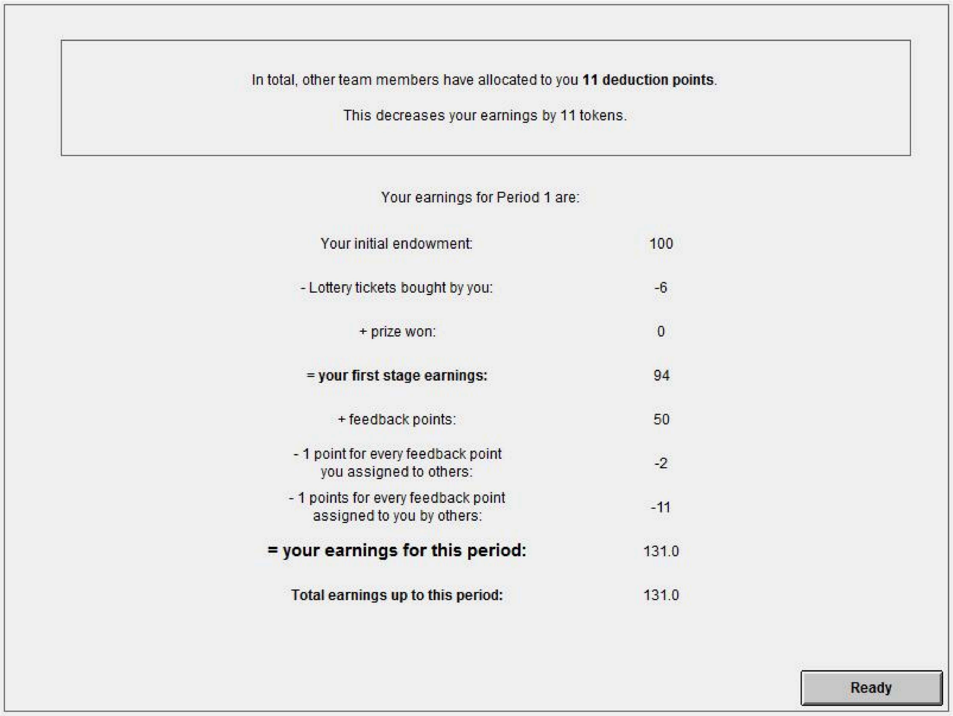

does not exist. Instead of lottery tickets for group M, blank tickets are generated. The amount of blank tickets corresponds to the Nash-equilibrium number for group M if it were still there. All lottery tickets are pooled and one ticket is randomly drawn. Each player belonging to the winning group earns the same prize z. If the winning ticket is blank, the prize is forfeited. In the following sections we analyse the single shot game and therefore omit a time index.

Single-shot Nash-equilibrium

Player $l \in K$ maximises the expected profit function

$$\pi_l(v) = \frac{v_l + \sum_{k \in K \setminus \{l\}} v_k}{v_l + \sum_{k \in K \setminus \{l\}} v_k + \sum_{m \in M} v_m} \cdot z - v_l.$$

Deriving the first order condition delivers the best response function for any player l of group K:

$$\frac{\partial \pi_l(v)}{\partial v_l} = 0 \quad \Leftrightarrow \quad v_l = \sqrt{z \cdot \sum_{m \in M} v_m} - \sum_{m \in M} v_m - \sum_{k \in K \setminus \{l\}} v_k$$

Checking the second order condition confirms that we find a maximum:

$$-\frac{2 \sum_{m \in M} v_m \cdot z}{\left(\sum_{k \in K} v_k + \sum_{m \in M} v_m \right)^3} < 0$$

Using the first order conditions for all players, we find a multiplicity of equilibria, characterised by

$$\sum_{k \in K} v_k = \frac{z}{4} \qquad \text{and} \qquad \sum_{m \in M} v_m = \frac{z}{4}.$$

S4 Fig plots the best response functions of both groups. If we assume symmetry in own group we get $v_l = \frac{z}{16}$.

Team reasoning strategy

Each team maximises expected earnings for the own group. Let v_K be the sum of expenditures of players $k \in K$ of own group K, while v_M denotes the sum of expenditures by players $m \in M$. The payoff function is

$$\pi_K(v) = \frac{v_K}{v_K + v_M} \cdot 4z - v_K.$$

Deriving delivers the best response function

$$\frac{\partial \pi_K(v)}{\partial v_K} = 0 \quad \Leftrightarrow \quad v_K = 2\sqrt{z v_M} - v_M.$$

We plot the best response functions for each group in S5 Fig . We verify that the second order condition is negative and use the first order conditions of both groups. We find the equilibrium $v_K = z$ and $v_M = z$.

S4 Fig. Best response strategies at team level for Nash Equilibrium.

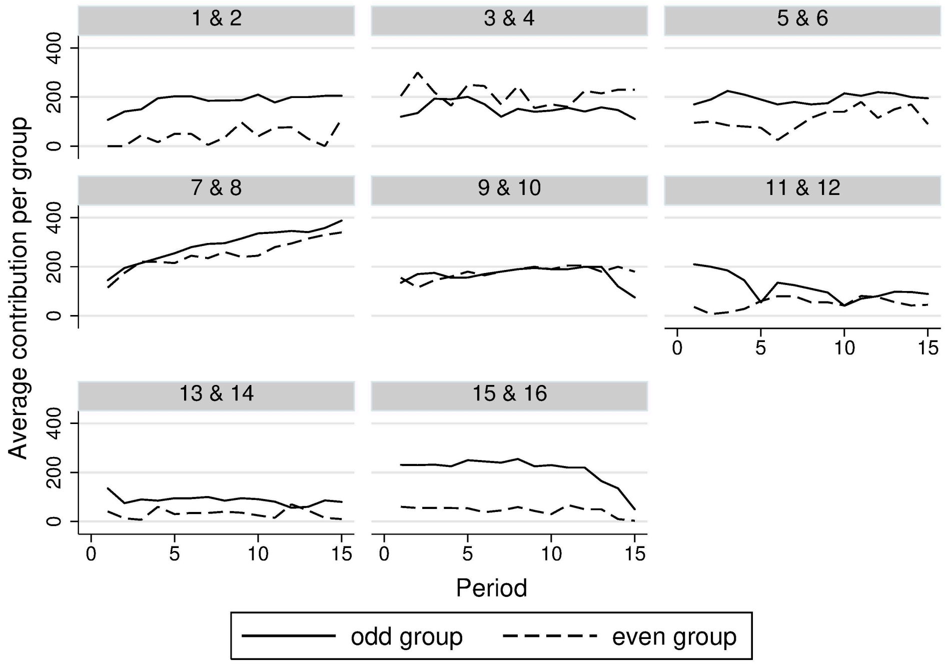

S5 Fig. Best response strategies at team level for Team reasoning strategy.

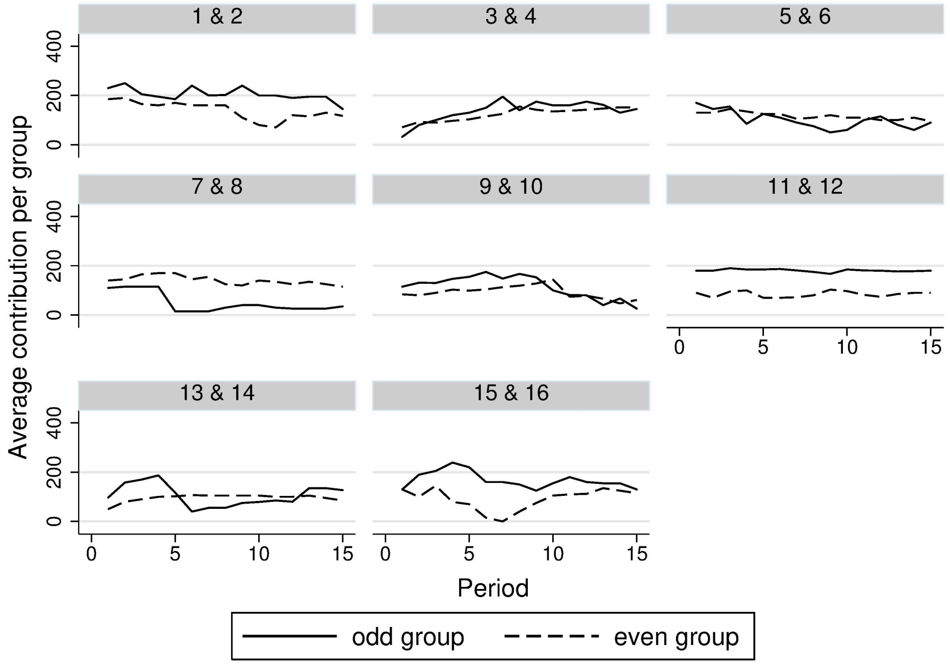

For the non-contest environment, the team reasoning strategy can be derived by substituting the contribution of group M in the best response function by the number of blank tickets which is the equilibrium quantity, i.e. $v_M = \frac{z}{4}$. This results in an equilibrium group contribution of $v_K = \frac{3}{4}z$.

Social optimal strategy: Non-contest environment

The socially optimal strategy in the non-contest environment coincides with the team reasoning strategy: $v_K = \frac{3}{4}z$.

Symmetric Nash-equilibrium with other-regarding preferences

We extend the model by very rudimentary other-regarding preferences. Players take into account the average payoffs of others in their own group K as well as in the other group M and give them weights θ_K^C and θ_M^C (superscript C stands for the contest environment). We assume the parameters to follow the restriction

$$1 > \theta_K^C > 0 > \theta_M^C > -1. \tag{7}$$

This means that players consider payoffs of their own group positively and payoffs of the other group negatively. The resulting utility function for player l is

$$u_l(v) = \pi_l(v) + \theta_K^C \cdot \frac{1}{3} \cdot \sum_{k \in K \setminus \{l\}} \pi_k(v) + \theta_M^C \cdot \frac{1}{4} \cdot \sum_{m \in M} \pi_m(v).$$

Deriving the first order condition delivers the best response function for any player l of group K:

$$\frac{\partial u_l(v)}{\partial v_l} = 0 \quad \Leftrightarrow \quad v_l = \sqrt{(1 + \theta_K^C - \theta_M^C) \cdot z \cdot \sum_{m \in M} v_m} - \sum_{m \in M} v_m - \sum_{k \in K \setminus \{l\}} v_k \tag{8}$$

Checking the second order condition confirms that we find a maximum given Assumption (7):

$$-\frac{2\left(1 + \theta_K^C - \theta_M^C\right) \sum\limits_{m \in M} v_m \cdot z}{\left(\sum\limits_{k \in K} v_k + \sum\limits_{m \in M} v_m\right)^3} < 0$$

Using the first order conditions for all players, we find a multiplicity of equilibria, characterised by

$$\sum_{k \in K} v_k = \left(1 + \theta_K^C - \theta_M^C\right) \cdot \frac{z}{4}.$$

Assuming symmetry, the individual investment is: $v_l = \left(1 + \theta_K^C - \theta_M^C\right) \frac{z}{16}$.

For the non-contest we allow the others' average payoff to be taken into account (weight θ_K^N, superscript N stands for non-contest) but neither as positively as the own group in the contest environment nor as negatively as the other group in the contest environment, i.e.

$$\theta_K^C > \theta_K^N > \theta_M^C. \tag{9}$$

The resulting utility function is

$$u_l(v) = \pi_l(v) + \theta_K^N \cdot \frac{1}{3} \cdot \sum_{k \in K \setminus \{l\}} \pi_k(v).$$

Under the assumption that the number of blank tickets is $\frac{z}{4}$ we can derive the best response function from the first order condition:

$$\frac{\partial u_l(v)}{\partial v_l} = 0 \quad \Leftrightarrow \quad v_l = \left(2\sqrt{1+\theta_K^N} - 1\right) \cdot \frac{z}{4} - \sum_{k \in K \setminus \{l\}} v_k$$

This leads to a multiplicity of equilibria which are characterized by

$$\sum_{k \in K} v_k = \left(2\sqrt{1+\theta_K^N} - 1\right) \cdot \frac{z}{4}.$$

From the equilibrium characterisations and Assumptions (7) and (9) we can verify that the group investments in the contest environment are higher than in the non-contest environment and also higher than in the plain equilibrium (in the absence of other-regarding preferences). If we further assume that $\theta_K^N > 0$, an assumption which is in line with the overwhelming majority of the literature, we can also conclude that the investments in the non-contest environment are higher with other-regarding preferences than without.

Group wise analysis of contribution

S6–S13 Figs depict average contribution per player across each of the 15 periods for each group. In S6–S9 Figs, odd-numbered group always plays against a group with a number that is one higher than the own one. The opposite is the case for even-numbered groups. This means that, for example, group 1 plays against group 2 or group 12 against group 11. As mentioned before, there has been no session of a group 3 & 4 in the baseline contest and group 6 in the baseline non-contest treatments, for why they are left blank.

S6 Fig. Average contribution per group, reward treatment, contest environment. Paired groups are displayed together.

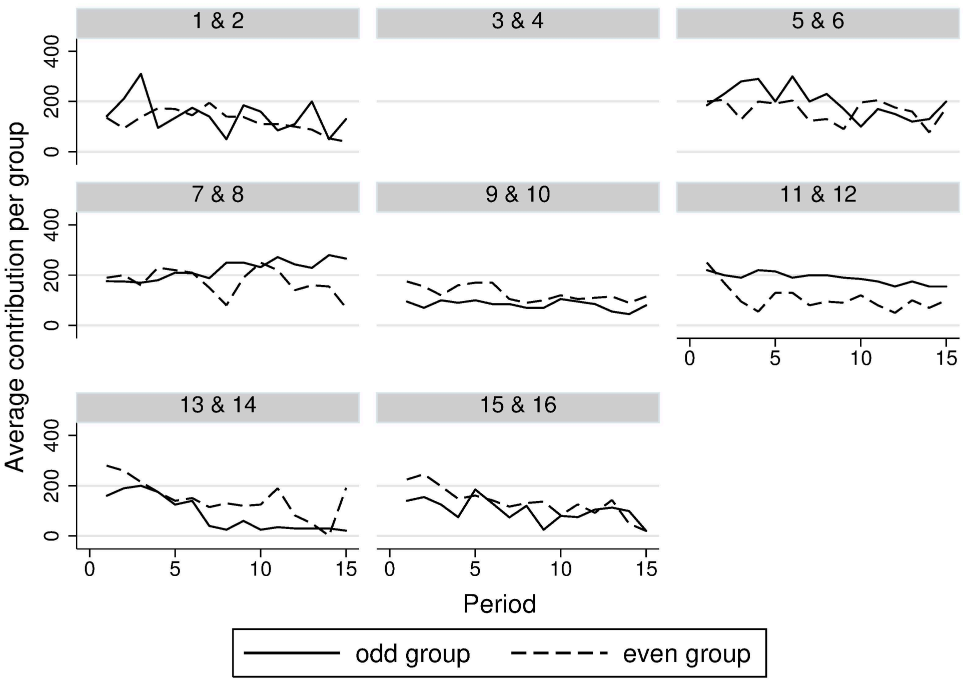

S7 Fig. Average contribution per group, punishment treatment, contest environment. Paired groups are displayed together.

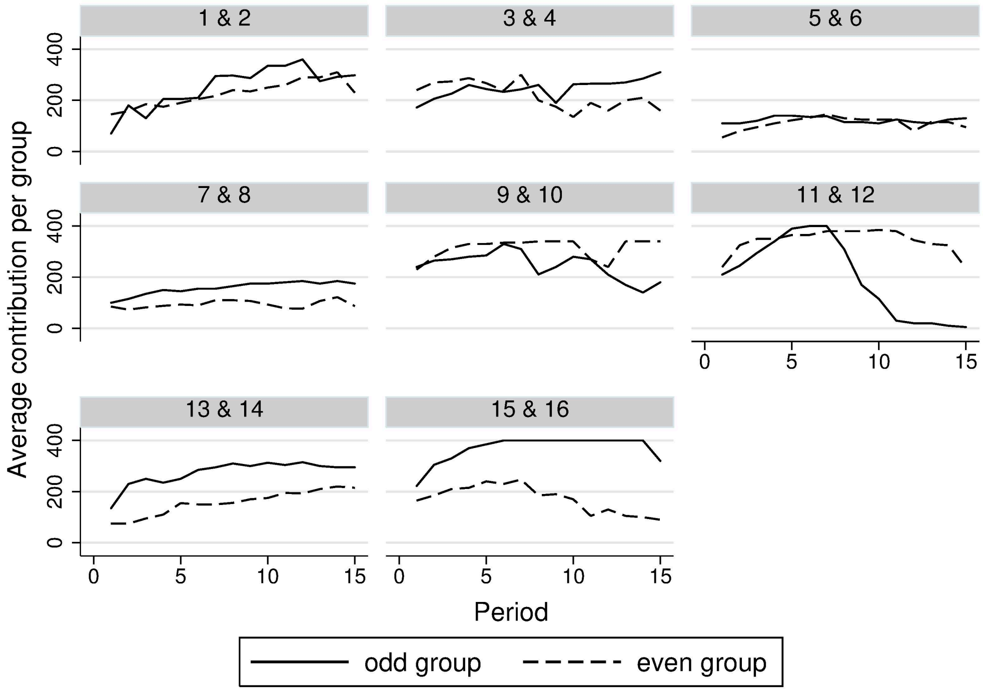

S8 Fig. Average contribution per group, baseline treatment, contest environment. Paired groups are displayed together. Session for groups 3 & 4 did not take place due to no-shows.

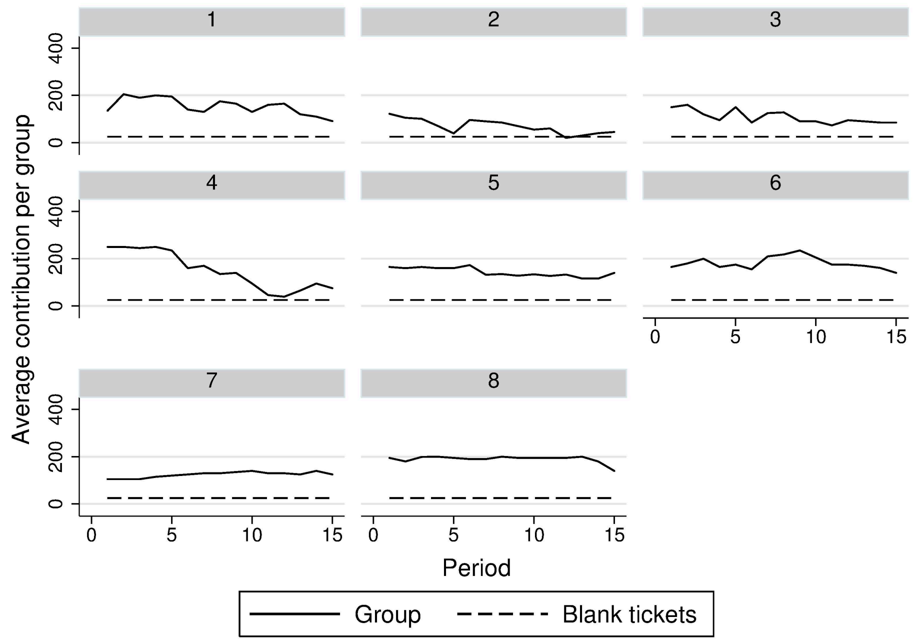

S9 Fig. Average contribution per group, R&P treatment, contest environment. Paired groups are displayed together.

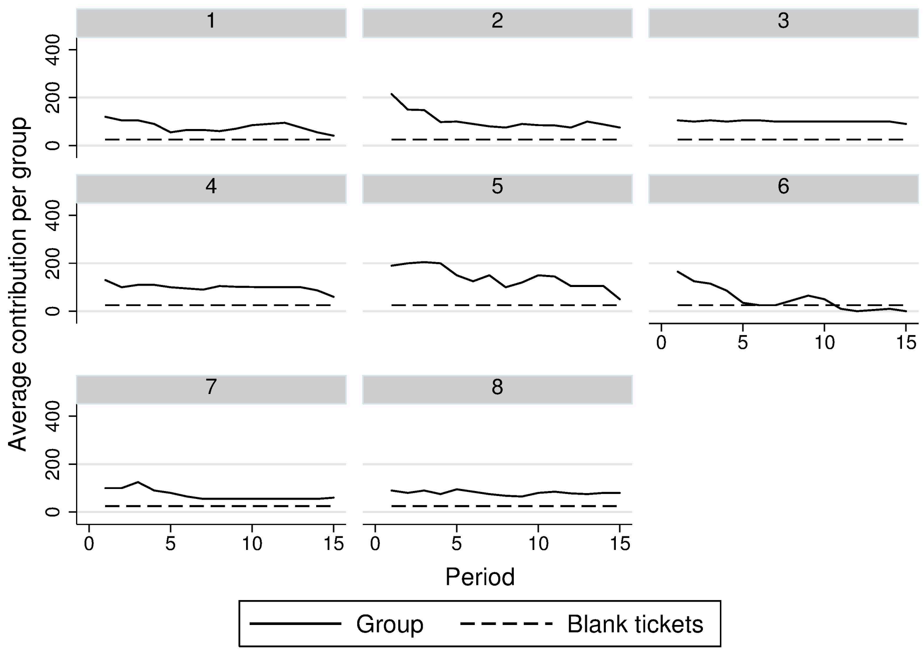

S10 Fig. Average contribution per group, reward treatment, non-contest environment.

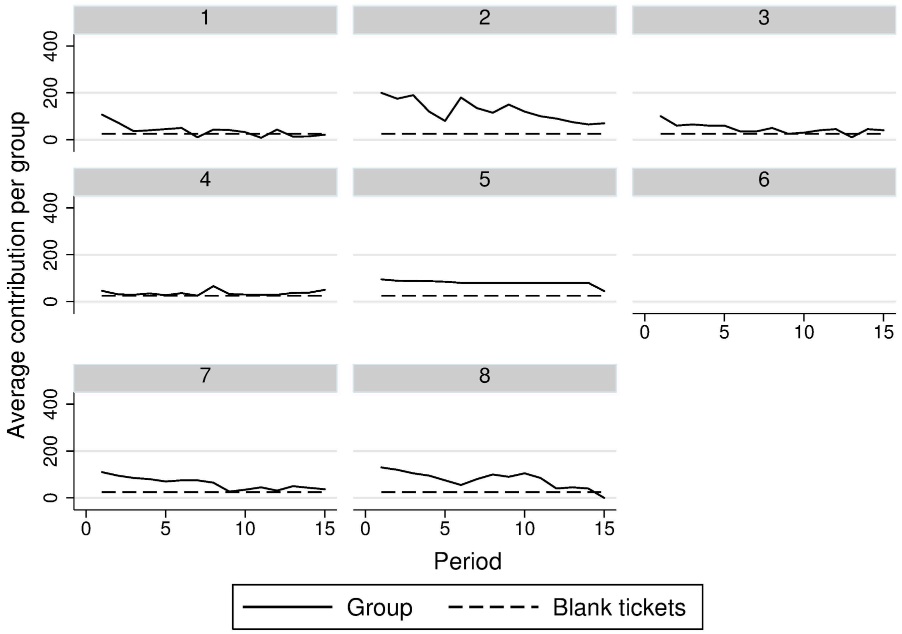

S11 Fig. Average contribution per group, punishment treatment, non-contest environment.

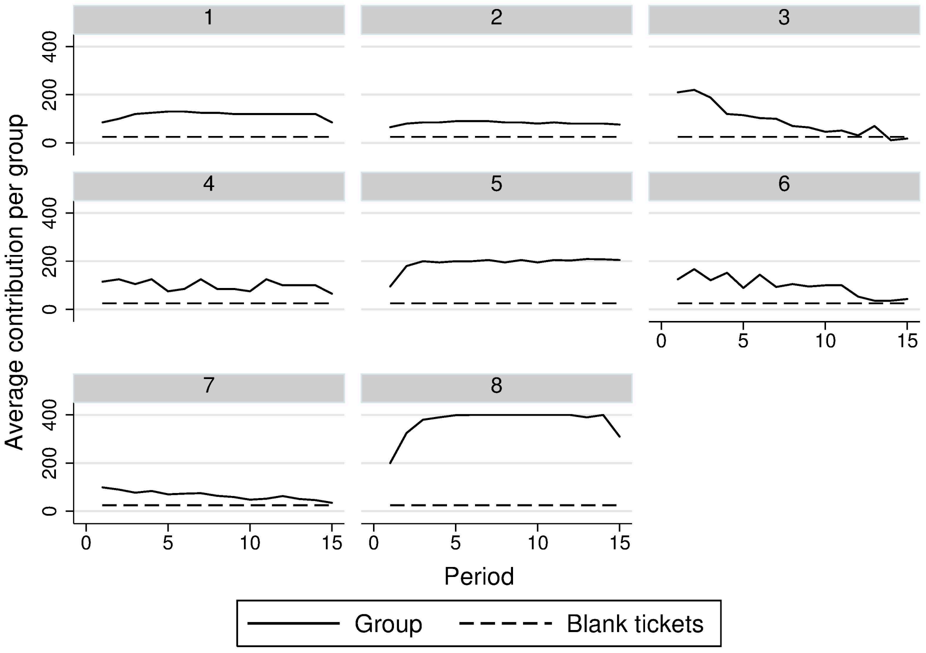

S12 Fig. Average contribution per group, baseline treatment, non-contest environment. Session for group 6 did not take place due to no-shows.

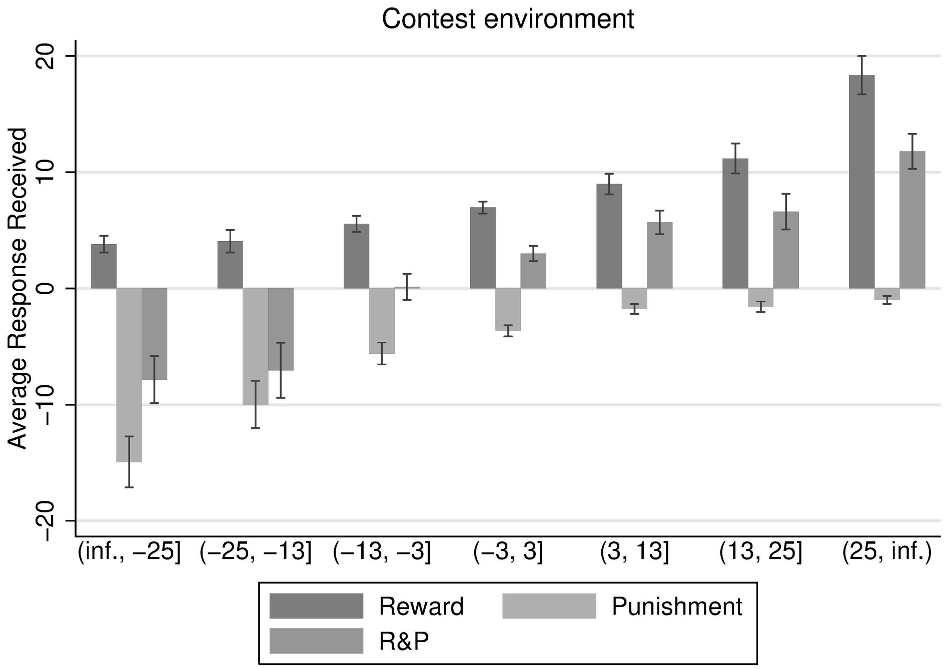

S13 Fig. Average contribution per group, R&P treatment, non-contest environment.

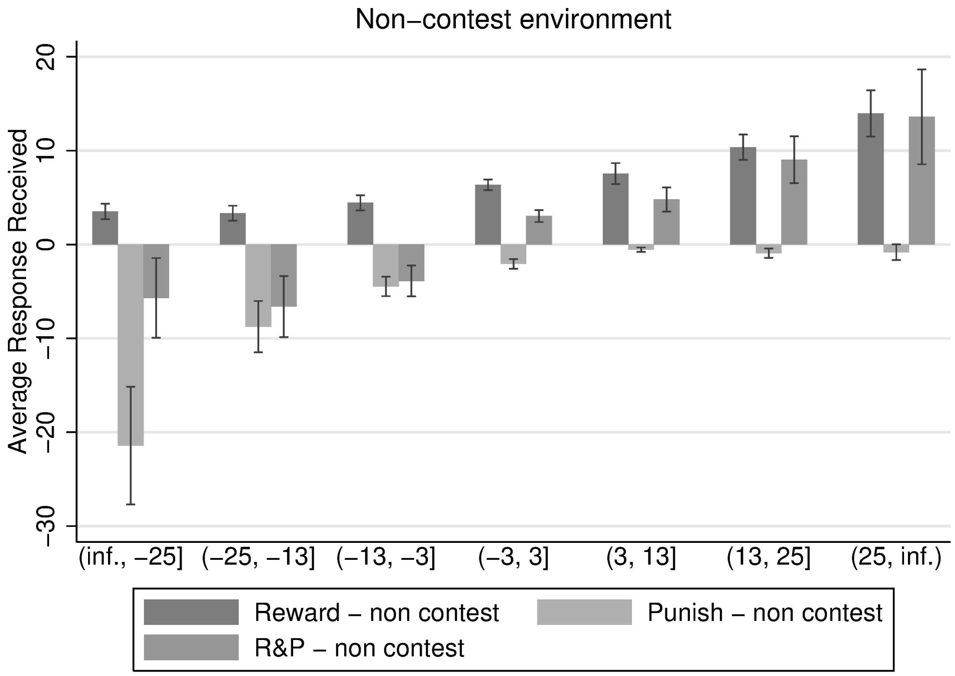

Personal attributes

S1 Table corresponds to regressions (10) and (12) from Table 6 with explicit control parameters. They were generated by participants' answers to a post-experiment questionnaire, in which we ask about a few personal features and stances. For this, we used questions from the World Values Survey [55]. In this section we examine the control factors in more detail.

Most notably, domestic students from the Netherlands and Belgium contribute substantially less to the contest than their colleagues from other countries do. This is the only significant country effect and its magnitude is somewhat impressive.

Players who state to place more importance on individual responsibility (as opposed to governmental responsibility) contribute less to the contest, but give more response. This factor was created on a scale from one to seven where individuals stated their proximity to which of the two statements they feel closer. Players with a higher preference for individual responsibility hence tend to be reluctant towards spending their money for the group project, but are willing to reciprocate by the means of response. "Preference for working alone" was also created on a scale of one to seven with the statement that the individual prefers working in groups or alone at the extremes, to which she expresses proximity. Players who prefer to work alone give less response, which means they act less reciprocal.

"Smoking" influences response-giving positively. We enquire on our participants' smoking behaviour as proxy for short-sightedness as in e.g. [56]. Next to a more reactionary response behaviour, individuals who indicate that they smoke also tend to spend less resources towards the contest.

Other items displaying a lowering effect towards contest spendings are "Family and friends important" (Participants' stated preference on a scale from 1—7) and "Politics important". They have been generated like the factor "Work important" on a seven point scale. The following two parameters, by contrast, display a positive effect on contest contributions: "Siblings" and "Study phase". Additionally, participants in a later "Study phase" use less tokens to reciprocate teammates' actions. Lastly, relatively older participants tend to spend less tokens to the contest, but more on reciprocating, which is reflected in regressor "Age". Given the participant body of our study is comprised almost exclusively of students, this result could open up an interesting pathway towards a follow-up study, employing a subject pool with a wider age structure.

Some factors for which we contemplated having explanatory power in this game remain toothless. "Current happiness" and "Ever practised a team sport", for example, neither have an influence on players' contest spendings nor response giving behaviour. Furthermore, the "Trust parameter" (Proximity to the two statements: Most people can be trusted—Need to be very careful) has no explanatory power towards participants' spending behaviour in the game. Placing tokens into the group account involves some degree of trust towards one's teammates that they will not renege or pursue an abating strategy. Lastly, "Work important" was created on a seven point scale in which participants express the importance of labour in their life.

Response received

This appendix presents additional steps of analysis in the context of Subsection Who receives Response?. First, S2 and S3 Tables present OLS regressions with error terms clustered on the

S1 Table. Individual level analysis.

VARIABLES	(10a)	(12a)
	Contribute	Own response
Contribute		0.268***
		(0.04)
Own response	0.815***	
	(0.14)	
Group contribution level (excl. self)	0.276***	−0.064***
	(0.02)	(0.01)
Group response level (excluding self)	−0.154**	0.143***
	(0.06)	(0.05)
Other group contribute	0.007	0.004
	(0.01)	(0.00)
Netherlands and Belgium	−6.756***	2.266
	(1.95)	(1.38)
Preference for working alone	0.176	−0.508
	(0.54)	(0.32)
Individual Responsibility	−1.226***	0.577**
	(0.46)	(0.26)
Work important	1.066	1.075
	(1.03)	(0.65)
Smoking	−4.160**	3.183**
	(2.01)	(1.53)
Trust parameter	0.878	0.824
	(1.32)	(0.76)
Family and friends important	−3.523***	0.181
	(1.13)	(0.82)
Politics important	−1.683*	0.198
	(0.91)	(0.46)
Current happiness	0.732	−0.681
	(1.18)	(0.54)
Ever practised a team sport	−0.430	0.980
	(1.28)	(0.82)
Siblings	1.312*	−0.499
	(0.73)	(0.32)
Study phase	3.649**	−3.760***
	(1.69)	(1.07)
Age	−0.935***	0.609***
	(0.30)	(0.22)
Constant	51.418***	−20.705**
	(12.88)	(8.14)
R-squared	0.749	0.426
N	270.000	270.000

* $p<0.05$,
** $p<0.01$,
*** $p<0.001$
Standard errors in parentheses. Study major control variables not reported.

group level of individual response received in period 1 on the player's level of contribution. Results are similar to regressions using data from the entire experiment. In period 1, however, the direction of causality is more clear-cut, as players have not received response in earlier rounds.

S2 Table. Contest environment. OLS regression with clustered error terms on group level.

	(21a)	(22a)	(23a)	(24a)
	**Reward**	**Punish**	**Reward R&P**	**Punish R&P**
**VARIABLES**	**Response received**			
Contribute	0.491***	−0.478**	0.043	−0.178
	(0.15)	(0.18)	(0.29)	(0.25)
Squared Contribute	−0.001	0.004*	0.003	0.001
	(0.00)	(0.00)	(0.00)	(0.00)
Constant	0.931	15.508***	13.784**	9.815*
	(1.32)	(4.34)	(5.82)	(5.59)

* $p<0.05$,
** $p<0.01$,
*** $p<0.001$
Standard errors in parentheses. NLB dummy and study major fixed effects not reported.

S3 Table. Non-contest environment. OLS regression with clustered error terms on group level.

	(25a)	(26a)	(27a)	(28a)
	**Reward**	**Punish**	**Reward R&P**	**Punish R&P**
**VARIABLES**	**Response received**			
Contribute	1.343***	−2.122**	1.181***	−1.017
	(0.28)	(0.77)	(0.27)	(0.56)
Squared Contribute	−0.010***	0.017**	−0.009*	0.011
	(0.00)	(0.01)	(0.00)	(0.01)
Constant	−14.110**	48.439**	−10.614***	24.072*
	(5.31)	(16.22)	(1.68)	(10.82)

* $p<0.05$,
** $p<0.01$,
*** $p<0.001$
Standard errors in parentheses. NLB dummy and study major fixed effects not reported.

S14 and S15 Figs present an alternative reference point for the response-giving decision, as compared to Figs 5 and 6. While the latter analysis depicts average response received contingent on individual deviation to the mean contribution level in the player's group, the Figures in this section consider a dyadic relationship instead. This means that here, the average response received is depicted in relation to the deviation from the sender's contribution in a given period. Results are discussed in Subsection Who receives Response?.

S14 Fig. Response received in relation to deviation from sender's contribution with 5% confidence interval.

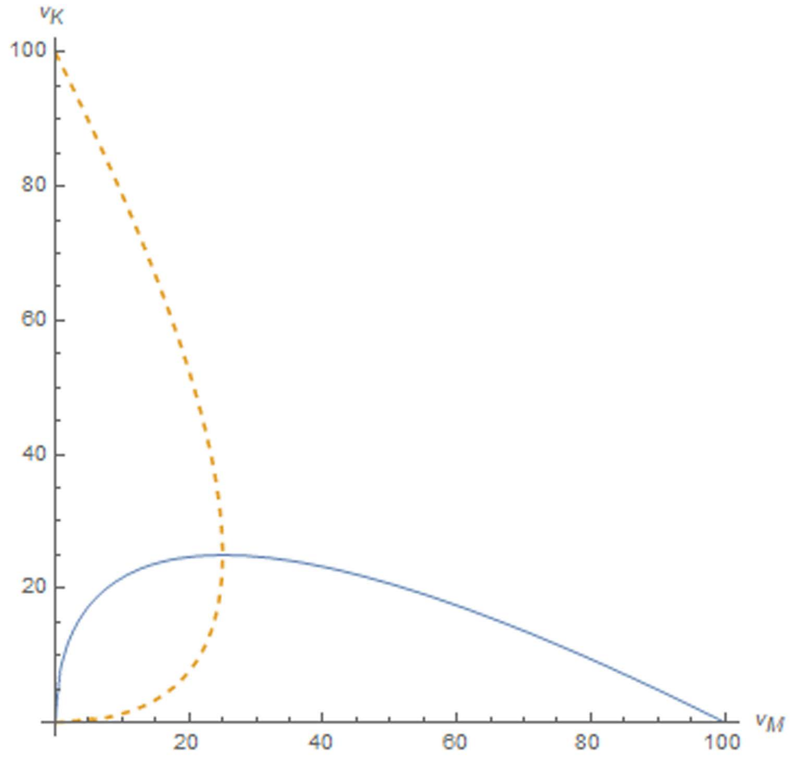

S15 Fig. Response received in relation to deviation from sender's contribution with 5% confidence interval.

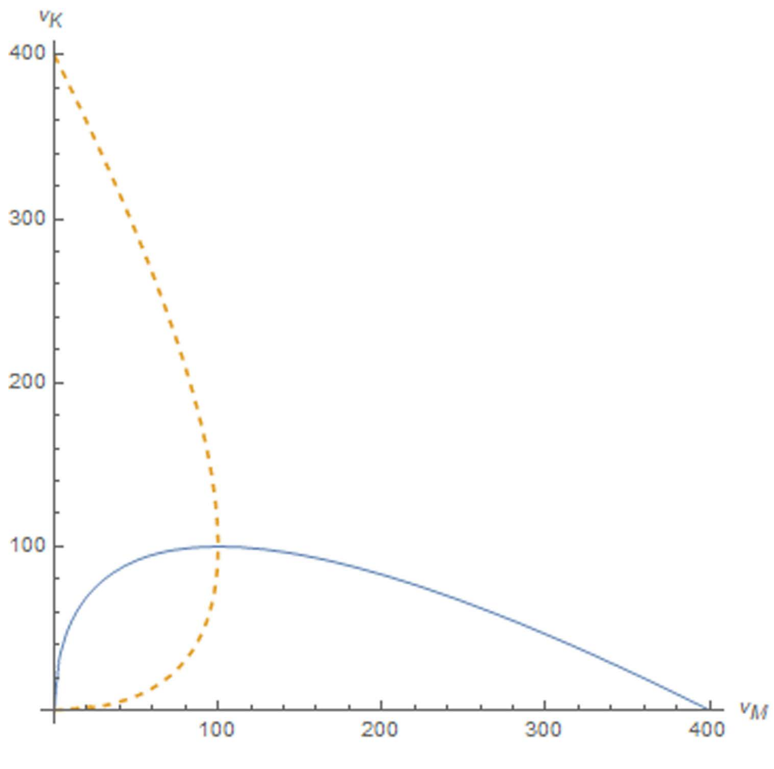

Example

Consider two players k and l, both members of the same group K in the R&P treatment, contest environment. Suppose they each spend $v_k = 40$ and $v_l = 5$ on lottery tickets in a given period. From the perspective of player k, the response she receives from l would be reflected in the rightmost bar of S14 Fig. The inverse, i.e. response from player k towards player l, would be represented by the third bar of the leftmost grouping.

The effect of winning

S4 Table. OLS regression with Newey-West standard errors.

VARIABLES	(29)
	**Contribute**
Won previous period (win1)	3.036
	(1.77)
Winning probability previous period (prob1)	34.981***
	(4.55)
Interaction term (win1×prob1)	−7.617**
	(2.95)
Constant	32.786***
	(3.62)

* p<0.05,
** p<0.01,
*** p<0.001
Standard errors in parentheses. Netherlands & Belgium, group and study major control variables not reported.
